# A surface-exposed GH26 β-mannanase from *Bacteroides ovatus*: Structure, role, and phylogenetic analysis of *Bo*Man26B

**DOI:** 10.1074/jbc.RA118.007171

**Published:** 2019-04-18

**Authors:** Viktoria Bågenholm, Mathias Wiemann, Sumitha K. Reddy, Abhishek Bhattacharya, Anna Rosengren, Derek T. Logan, Henrik Stålbrand

**Affiliations:** From the ‡ Department of Biochemistry and Structural Biology, Lund University P. O. Box 124, S-221 00, Lund, Sweden and; the § Department of Molecular Sciences, Swedish University of Agricultural Sciences Box 7015, 750 07, Uppsala, Sweden

**Keywords:** carbohydrate metabolism, structure–function, Gram-negative bacteria, phylogenetics, enzyme kinetics, glycoside hydrolase, Bacteroides ovatus, galactomannan, human gut bacteria, polysaccharide utilization loci

## Abstract

The galactomannan utilization locus (*Bo*ManPUL) of the human gut bacterium *Bacteroides ovatus* encodes *Bo*Man26B, a cell-surface–exposed endomannanase whose functional and structural features have been unclear. Our study now places *Bo*Man26B in context with related enzymes and reveals the structural basis for its specificity. *Bo*Man26B prefers longer substrates and is less restricted by galactose side-groups than the mannanase *Bo*Man26A of the same locus. Using galactomannan, *Bo*Man26B generated a mixture of (galactosyl) manno-oligosaccharides shorter than mannohexaose. Three defined manno-oligosaccharides had affinity for the SusD-like surface–exposed glycan-binding protein, predicted to be implicated in saccharide transport. Co-incubation of *Bo*Man26B and the periplasmic α-galactosidase *Bo*Gal36A increased the rate of galactose release by about 10-fold compared with the rate without *Bo*Man26B. The results suggested that *Bo*Man26B performs the initial attack on galactomannan, generating oligosaccharides that after transport to the periplasm are processed by *Bo*Gal36A. A crystal structure of *Bo*Man26B with galactosyl-mannotetraose bound in subsites −5 to −2 revealed an open and long active-site cleft with Trp-112 in subsite −5 concluded to be involved in mannosyl interaction. Moreover, Lys-149 in the −4 subsite interacted with the galactosyl side-group of the ligand. A phylogenetic tree consisting of GH26 enzymes revealed four strictly conserved GH26 residues and disclosed that *Bo*Man26A and *Bo*Man26B reside on two distinct phylogenetic branches (A and B). The three other branches contain lichenases, xylanases, or enzymes with unknown activities. Lys-149 is conserved in a narrow part of branch B, and Trp-112 is conserved in a wider group within branch B.

## Introduction

The human gut microbiota is important for our well-being due to its widespread implications associated with human health (1–4). These vital microbes, present in the colon, encode enzymes and other proteins responsible for capture and breakdown of dietary fibers, such as hemicellulosic polysaccharides (5, 6). The gut microbiota may change in response to our diet due to differences in the individual catabolic capabilities among the species (5, 7). In this study, we focus on the utilization mechanisms of the hemicellulosic polysaccharide galactomannan, known to be fermented in the human gut (8). A deeper understanding of the utilization of different dietary fibers by our gut microbes and the mechanisms involved will likely expand our possibilities to affect our microfloral balance through our diet. This could ultimately be beneficial to our health (9) and has been investigated for example with the β-fructan inulin (5, 10). Here we report on the galactomannan degradation machinery of the common human gut bacterium *Bacteroides ovatus*, with a focus on the extracellular, cell attached β-mannanase *Bo*Man26B (11) for which we here solve the crystal structure.

The Gram-negative Bacteroidetes is a dominant phylum in the human gut, encoding a large number of different glycoside hydrolases (GHs)^3^ for dietary fiber processing, often organized in gene clusters termed polysaccharide utilization loci (PULs) (6, 12). A PUL encodes GHs and other proteins required for recognition, binding, degradation, and internalization of a specific type of polysaccharide (13–16). Although with large differences between species, Bacteroidetes are known to utilize many different polysaccharides and are therefore well-adapted to survival in the gut (7, 17).

β-Mannans are hemicellulosic polysaccharides, present in our diet as storage polysaccharides (18) and food thickeners (19, 20). β-Mannan has a β-1,4-linked linear backbone (21). In glucomannans, the mannan backbone is interrupted by glucose units (22). Galactomannans such as locust bean gum (LBG) and guar gum carry α-1,6-linked galactosyl side-groups (23).

To utilize β-mannans, several different types of GHs are required: β-1,4-mannanases and β-1,4-mannosidases cleave the mannan backbone and α-1,6-galactosidases and esterases remove side-groups (24). The GHs are classified into different families based on sequence similarity in the carbohydrate-active enzyme (CAZy)^4^ database (25). β-Mannanases are found in glycoside hydrolase families GH5, GH26, GH113, and GH134. GH5, -26, and -113 belong to clan GH-A composed of enzymes that have a (β/α)_8_-barrel fold and catalyze glycosidic bond hydrolysis through a conserved retaining mechanism with two catalytic residues, a nucleophile (Glu) and an acid/base (Glu) (24). The saccharide is typically bound in an active-site cleft containing several subsites for sugar binding, numbered from the nonreducing end (−2, −1, +1, +2, etc.), with bond-cleavage occurring between subsite −1 and +1 (26). GH26 β-mannanases characterized so far generally show decreased activity toward β-mannans with increasing levels of galactose side-groups (27–31). GH26 primarily contains bacterial β-mannanases, with relatively few determined crystal structures (11, 32–39). The only previous GH26 structure that originates from the gut microbiota is that of *Bo*Man26A of *B. ovatus* (11), and only a few β-mannanases from this GH-rich microbiome have been characterized (11, 30, 40, 41).

**Figure 1.**
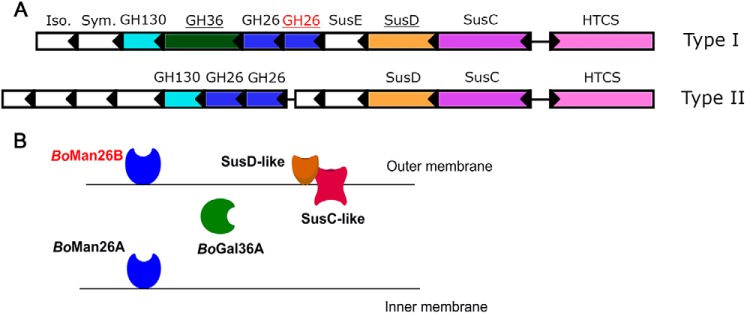
*A,* overview of the two types of GH26-encoding PULs identified in *Bacteroides* spp. by Reddy *et al.* (43) showing *Bo*ManPUL at the top (type I). The genes are colored and labeled according to putative function. Genes encoding for a putative isomerase (*Iso.*), symporter (*Sym.*), and SusE-positioned protein are labeled. The functions of the HTCS-type regulator, SusD-like protein, GH26 and GH36 enzymes have been experimentally confirmed in the type I *Bo*ManPUL (11, 16). The genes encoding proteins studied in this work are *underlined* with the gene encoding *Bo*Man26B labeled in *red. B,* schematic overview showing the location of the *Bo*ManPUL-encoded proteins studied in this paper (adapted from Bagenholm *et al.* (11)).

The *B. ovatus* strain ATCC 8483 contains several PULs for hemicellulose utilization (16, 42). The β-mannanases *Bo*Man26A and *Bo*Man26B are encoded by one of these, which is a galactomannan PUL (11, 43) (gene locus bacova_02087–97, hereafter termed “*Bo*ManPUL”) (Fig. 1). *Bo*ManPUL also codes for a hybrid two-component system (HTCS)–like regulator that binds manno-oligosaccharides (16), a GH36 α-galactosidase (*Bo*Gal36A) (43), and several proteins (11) similar to those encoded by the archetypical starch PUL from *Bacteroides thetaiotaomicron,* examples being outer-membrane glycan–binding (SusD) and transport (SusC) proteins (13). We have identified variations among the predicted *Bacteroides* mannan PULs with partial homology to *Bo*ManPUL, all encoding two predicted GH26 mannanases (Fig. 1*A*) (43). Type I PULs also encode an α-galactosidase (GH36), but type II PULs do not.

Our previous study led to the following model of galactomannan utilization conferred by *Bo*ManPUL-encoded proteins (11). Extracellular *Bo*Man26B initially cleaves galactomannan into shorter oligosaccharides, which are transported into the periplasm by the SusC-like transporter (Fig. 1*B*). In the periplasm, *Bo*Gal36A removes the galactose side-groups, and then *Bo*Man26A cleaves the manno-oligosaccharides to mannobiose, which is imported to the cytosol (11, 43). During our previous study of *Bo*ManPUL proteins (11), several significant differences were observed between the GH26 β-mannanases. *Bo*Man26A is periplasmic, more active on oligosaccharides, and severely restricted by galactose side-groups, whereas the partially characterized *Bo*Man26B is associated with the outer membrane, with very low sensitivity to galactose side-groups and a preference for longer substrates (11). The crystal structure of *Bo*Man26A shed light on its mode of substrate attack (11), something which is hitherto lacking for *Bo*Man26B.

The major aim of this study was to structurally and functionally characterize the outer membrane β-mannanase *Bo*Man26B to further elucidate its role in the *Bo*ManPUL galactomannan degradation machinery. In this work, we present the crystal structure and functional properties of *Bo*Man26B, which were shown to be distinctly different compared with the previously characterized periplasmic β-mannanase *Bo*Man26A (30% identity) expressed from the same PUL (11). To expand the knowledge on the GH26 enzymes, a phylogenetic tree was generated, and our results indicate that at least some other *Bacteroides* harbor a similar pair of GH26 β-mannanase genes present in PULs, which points toward PUL-encoded β-mannanase diversity also among other species.

## Results

### Galactomannan kinetics of BoMan26B

Unlike several other GH26 β-mannanases (27–31), *Bo*Man26B shows a high tolerance for galactose side-groups (11). Michaelis-Menten kinetics for *Bo*Man26B on LBG and the more highly galactose-substituted guar gum revealed a 60% lower apparent *k*_cat_/*K_m_* for guar gum compared with LBG. This was due to a 2-fold higher *k*_cat_ on LBG but similar *K_m_* values (Fig. S1 and Table 1). *Bo*Man26B is less efficient at hydrolyzing LBG, with 5–10 times lower *k*_cat_/*K_m_*, compared with several other GH26 β-mannanases (29, 31, 44). However, *Bo*Man26B *k*_cat_/*K_m_* was only reduced by about 60% on guar gum compared with LBG (Table 1 and Fig. S1). The large reduction in *k*_cat_/*K_m_* for some other GH26 mannanases (29, 45) is due to an increased *K_m_*, whereas for *Bo*Man26B the *K_m_* remained similar. This further signifies that *Bo*Man26B is more tolerant for galactosyl side-groups carried by galactomannan compared with other characterized GH26 β-mannanases.

**Table 1:** **Polysaccharide kinetics for *Bo*Man26B and the variants K149S and K149A** The data include the estimation of *k*_cat_/*K_m_* for the variants K149S, K149A, W112F, and W112A using linear regression for LBG and guar gum.

	*K_m_*	*k*_cat_	*k*_cat_/*K_m_^a^*
	g/liter	s^−1^	g/liter^−1^s^−1^
**LBG**			
*Bo*Man26B	10.7 ± 1.1	250 ± 12.1	23.3 ± 2.7
K149S	21.6 ± 4.5	179 ± 23.1	8.24 ± 2.0
K149A	20.1 ± 3.7	178 ± 14.1	8.68 ± 1.7
W112F			1.2
W112A			0.98
**Guar gum**			
*Bo*Man26B	12.9 ± 1.7	122 ± 9.4	9.5 ± 1.4
K149S			3.2
K149A			3.6
W112F			0.71
W112A			0.56

*^a^* The *k*_cat_/*K_m_* values for classical Michaelis-Menten kinetics are in agreement with the estimated *k*_cat_/*K_m_* value based on linear regression.

### Product length of BoMan26B

**Figure 2.**
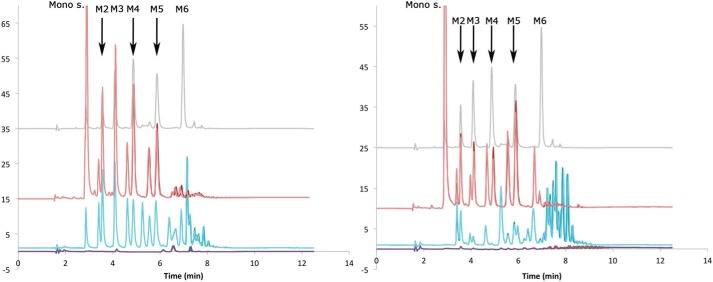
**Determination of the product length of *Bo*Man26B using LBG (*left*) or guar gum (*right*).** Duplicates of the blank (*purple*), incubation with *Bo*Man26B (*blue*), and after treatment of hydrolysis products with guar α-galactosidase (*red*) are shown as well as a standard with 2.5 μm M1–M6 (*gray*). The monosugar (galactose and mannose) and M2–M6 peaks are indicated.

To determine the length of end products, *Bo*Man26B was incubated with LBG and guar gum galactomannan for 24 h. Samples were analyzed with high-performance anion-exchange chromatography with pulsed amperometric detection (HPAEC-PAD), showing a complex mixture of oligosaccharide products (Fig. 2), many of which are likely galactosylated. To enable determination of the product's degree of polymerization (DP), the hydrolysates were treated with guar α-galactosidase (46) to cleave off galactosyl side-groups. After the treatment, a shift of the chromatogram was observed: peak areas beyond DP5 were severely reduced, whereas the monosaccharide and mannobiose to mannopentaose (M2–M5) peak areas increased for both substrates (Fig. 2). After treatment with guar α-galactosidase, the primary products were revealed to be M2 to mannotetraose (M4) from LBG and M5 from guar gum (Fig. 2). The range of manno-oligosaccharides generated by *Bo*Man26B fits with its previously proposed role as the enzyme responsible for initial endo-attack on galactomannan (11).

### Synergy of BoMan26B and BoGal36A

Synergy between *Bo*Man26B and *Bo*Gal36A, the subsequent GH in the proposed galactomannan utilization pathway (11), was assessed by quantifying release of M2 and galactose from LBG and guar gum galactomannan. Upon co-incubation of *Bo*Man26B and *Bo*Gal36A, M2 release increased 2.5 times for LBG and 3.5 times for guar gum, whereas the increase in galactose release was significantly larger at about 10 and 5 times higher, respectively (Table 2). The significantly higher increase in galactose release suggests a sequential enzyme synergy, where *Bo*Man26B acts first on the galactomannan, followed by *Bo*Gal36B. This view is supported by the cellular location of the enzymes, because *Bo*Man26B is extracellular and *Bo*Gal36A is periplasmic (11).

**Table 2:** **Synergy experiments using LBG and guar gum**

	M2 release	Gal release
	μ*m*/*min*	
**LBG**		
*Bo*Man26B	32.1 ± 0.68	ND*^a^*
*Bo*Gal36A	ND	5.33 ± 2.0
*Bo*Man26B + *Bo*Gal36A	74.2 ± 12.6	55.7 ± 7.9
**Guar**		
*Bo*Man26B	10.8 ± 0.03	ND
*Bo*Gal36A	ND	18.0 ± 4.6
*Bo*Man26B + *Bo*Gal36A	38.0 ± 8.2	86.8 ± 24.2

*^a^* ND means not detected.

### SusD-like protein sugar binding

The SusD-like protein encoded by the *Bo*ManPUL has affinity for galactomannan and glucomannan (11). To investigate potential affinity for manno-oligosaccharides, microscale thermophoresis (MST) analysis was performed using fluorescently labeled protein. Regular MST experiments, which require a stable fluorescence signal regardless of ligand concentration, could not be performed due to a significant decrease of signal with increasing concentrations of ligand (6^3^,6^4^-α-d-galactosyl-mannopentaose (G2M5), mannohexaose (M6), or M5). This signal decrease can arise from interactions due to specific binding of the ligand or unspecific interactions between the ligand and the protein or dye. If the decrease is due to specific interaction, the fluorescence data could be used to calculate the affinity of the SusD-like protein for the oligosaccharides, as has been done for other systems (47, 48). The SDS denaturation test (SD-test) restored the fluorescent signal, and there was no observable decrease in fluorescence with increasing ligand concentrations for G2M5 and the similarly labeled control protein *Thermotoga maritima* ribonucleotide reductase (*Tm*NrdD) (Fig. S2). Thus, the decrease in fluorescence with increasing ligand concentrations is likely due to specific interaction between G2M5, M6, or M5 and the SusD-like protein, which can be attributed to the changes in the environment of the fluorophore when ligand binding occurs.

**Figure 3.**
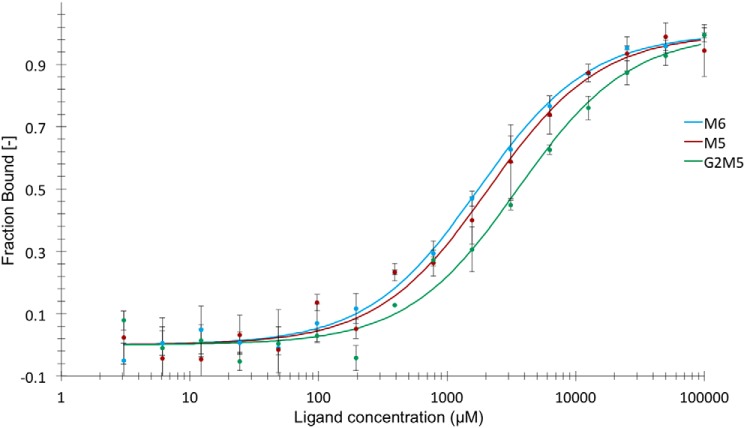
**MST analysis of binding of SusD-like protein to G2M5 (*green*), M6 (*blue*), and M5 (*red*).** The binding constant, *K_D_*, was calculated from the fitted curve for respective saccharide. The *error bars* show the standard deviation for each point.

The *K_d_* values for the interactions between the SusD-like protein and G2M5, M6, and M5 were 3.5 ± 0.6, 1.8 ± 0.2, and 2.1 ± 0.3 mm, respectively (Fig. 3). The binding of SusD-like protein to manno-oligosaccharides with a main chain DP of 5–6 agrees with the production of DP2–5 oligosaccharides by *Bo*Man26B from galactomannan substrates (Fig. 2).

### Structure of BoMan26B

**Figure 4.**
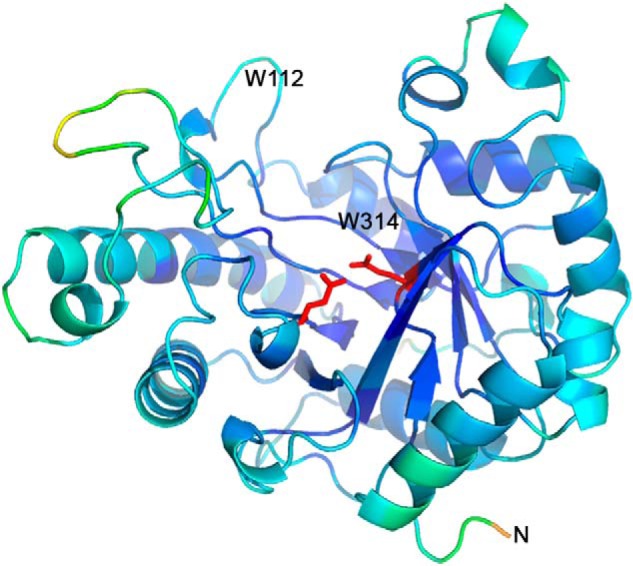
**Overview of *Bo*Man26B looking into the active-site cleft colored according to the B-factor between 10.5 Å^2^ (*dark blue*) to 68.7 Å^2^ (*red*).** The catalytic residues (*red*) are shown in *stick* representation. The positions of the residues Trp-112 and Trp-134, as well as the N terminus, have been labeled.

To investigate the structural basis for the galactose side-group tolerance of *Bo*Man26B, and the biochemical differences observed with *Bo*Man26A, two crystal structures of *Bo*Man26B were obtained: an apoenzyme structure and a structure containing the saccharide 6^3^-α-d-galactosyl-mannotetraose (G1M4) in the active-site cleft. Both structures had one monomer in the asymmetric unit (residues 37–361 were possible to model in both cases) and were collected at 1.7 Å (apoenzyme structure) or 1.8 Å (G1M4 complex structure) resolution (Table 3, PDB codes 6HF2 and 6HF4, respectively). The two structures are very similar, 297 Cα atoms align with 0.148 Å root mean square deviation (RMSD), using PyMOL (49). The main difference is an ordering of Lys-149 by the −4 subsite in the G1M4 complex structure. As for other GH26 β-mannanases with determined structures (11, 32–37, 39), *Bo*Man26B has a (β/α)_8_-barrel fold with an additional N-terminal α-helix (Fig. S3) and the two catalytic residues (predicted acid/base Glu-201 and nucleophile Glu-291 (50)) located in a cleft (Fig. 4). The ligand soaking conditions contained a mixture of G2M5, 6^1^-α-d-galactosyl-mannotriose (GM3), mannotriose (M3), and 6^1^-α-d-galactosyl-mannobiose (GM2), none of which correspond to the G1M4 seen the in the active-site cleft. The observed oligosaccharide may thus represent a contaminant or, less likely, be the result of a combination of the shorter soaked substrates (GM2 and M3). A weak electron density was seen in the −1 and +1 subsites of the apoenzyme structure, but no molecule from expression, purification, or crystallization could be modeled with confidence.

**Table 3:** **Data collection and refinement statistics** Statistics for the highest resolution shell shown in parentheses.

	Apoenzyme structure	G1M4 complex structure
Resolution range (Å)	47.79–1.69 (1.75–1.69)	44.33–1.78 (1.85–1.78)
Space group	P2_1_2_1_2_1_	P2_1_2_1_2_1_
Unit cell (Å, °)	66.81, 68.38, 79.14, 90, 90, 90	50.12, 68.36, 95.00, 90, 90, 90
Total reflections	504,797	284,275
Unique reflections*^a^*	40,664 (3477)	57,377 (4265)
Completeness (%)	98.5 (84.9)	95.1 (70.1)
Multiplicity	12.4 (7.8)	5.0 (3.2)
〈*I*〉/〈σ(*I*)〉	18.2 (2.5)	6.3 (0.9)
*R*_merge_ (*I*)	0.101 (0.713)	0.152 (0.81)
*CC*_½_	1.00 (0.89)	0.99 (0.51)
Wilson *B*-factor (Å^2^)	21.3	24.6
*R*-work	0.166 (0.329)	0.178 (0.293)
*R*-free	0.207 (0.341)	0.210 (0.338)
No. of nonhydrogen atoms	3010	2937
Macromolecules	2659	2676
Associated atoms and ligands*^b^*	2	58
Water molecules	350	203
Modeled protein residues	325	325
RMSD*^c^* (bonds, Å)	0.009	0.007
RMSD*^c^* (angles, °)	0.95	0.87
Ramachandran favored (%)	96.6	96.9
Ramachandran outliers (%)	0	0.62
Clashscore*^d^*	3.3	3.0
Average *B*-factor (Å^2^)	23.5	28.1
Macromolecules	22.3	27.3
Associated atoms and ligands*^b^*	24.8	40.7
Water	32.6	34.8

*^a^* The number of nonanomalous unique reflections are shown.

*^b^* This includes a chloride ion and a calcium ion for both structures and a bound G1M4 ligand for the G1M4 complex structure.

*^c^* RMSD is root mean square deviation from ideal geometry.

*^d^* Unfavorable all-atom steric overlaps are ≤0.4 Å per 1000 atoms (72).

### Metal-binding site in BoMan26B

*Bo*Man26B contains a potential calcium-binding site and was 100% stable over 24 h at 37 °C in the presence of 1 mm calcium, but it lost about 40% activity when it was not included. A calcium ion was modeled with 0.5 occupancy in the apoenzyme structure coordinated by Ser-108, Leu-105, and Glu-179 (Fig. S4) and three water molecules with a binding geometry typically seen for calcium. Higher calcium concentrations (50 mm compared with 0.3 mm in the apoenzyme structure) resulted in full occupancy in the G1M4 complex structure (Fig. S4). The calcium-binding site is not located in the active-site cleft and thus does not have a glycan-binding role. Stabilizing metal sites have previously been seen in a few thermostable β-mannanases from GH5 and GH26 (35, 51–53); however, these sites are not conserved with *Bo*Man26B.

### Binding of the G1M4 saccharide in the active-site cleft of BoMan26B

**Figure 5.**
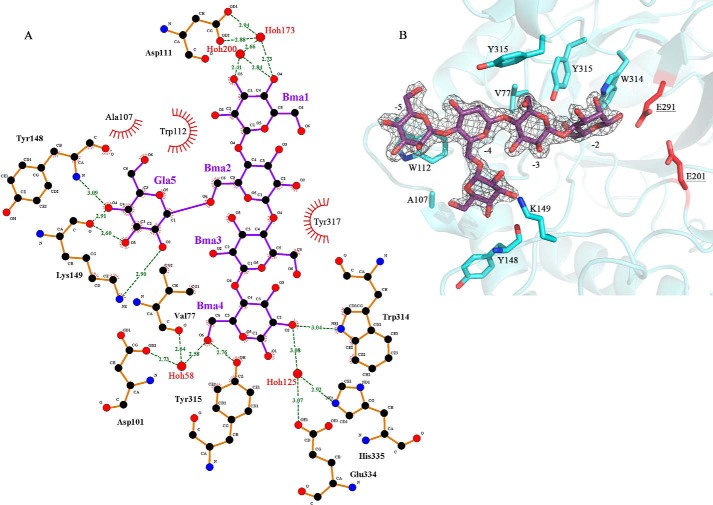
**Glycan interaction in *Bo*Man26B.** *A,* interactions with the sugar residues from the G1M4 complex structure bound in subsites −5 (Bma1) to −2 (Bma4), with a galactose side-group (Gla5) in the −4 subsite (Bma2), shown using LigPlot (76). Interactions bridged by water molecules are included. Hydrogen bond distances are labeled, and hydrophobic interactions are shown as *red fans. B,* 2m|*F_o_*| − D|*F_c_*| electron density for the G1M4 ligand, contoured at 1.0 σ. The bound saccharide and all residues shown to interact directly with the G1M4 complex oligosaccharide in *A* are shown with *stick* representation, and the residues are labeled. The catalytic residues (*red*) are also shown for context.

A G1M4 oligosaccharide could be modeled in subsites −5 to −2 of the active-site cleft (Fig. 5). The mannosyl unit in the −5 subsite stacks with Trp-112 and is involved in a hydrogen bonding network with Asp-111 (Table 4 and Fig. 5). Accommodation of galactose side-groups is likely in this subsite as the closest residue to the mannosyl group O6 is the OH of Tyr-317 (5.7 Å). Subsite −4 has no clear interactions with the backbone mannosyl group, but the attached galactose unit hydrogen bonds to Tyr-148 and Lys-149 in *Bo*Man26B (Table 4). This indicates that a side-group may be favored in this subsite.

**Table 4:** **Interactions between *Bo*Man26B and the G1M4 ligand bound in subsites −5 to −2**

Subsite	Saccharide unit	Saccharide atom	H-bond distance (including intermediate waters)	Residue atom	Interacting residue
−5	Man		Stacking		Trp-112
		O3	2.4 Å w200–2.7 Å–w173–2.9 Å	OD1	Asp-111
		O3	2.4 Å–w200–2.7 Å–w173–2.9 Å	OD2	Asp-111
		O4	2.7 Å–w173–2.9 Å	OD1	Asp-111
		O4	2.7 Å–w173–2.9 Å	OD2	Asp-111
−4*^a^*	Gal	O2	2.9 Å	NZ	Lys-149
		O3	2.6 Å	O	Tyr-148
		O4	3.1 Å	N	Tyr-148
		O4	2.9 Å	O	Tyr-148
		O6	3.1 Å–w114–2.9 Å	OD1	Asn-104
		O6	3.1 Å–w114–3.1 Å	N	Ala-107
−3	Man		Stacking		Tyr-317
−2	Man	O2	3.0 Å	NE1	Trp-314
		O2	3.1 Å–w125–3.1 Å	OE1	Glu-334
		O2	3.1 Å–w125–2.9 Å	NE2	His-335
		O6	2.8 Å	OH	Tyr-315
		O6	2.6 Å–w58–2.6 Å	O	Val-77
		O6	2.6 Å–w58–2.7 Å	OD2	Asp-101

*^a^* Data show the interactions of the galactose side group for the mannosyl unit occupying the −4 subsite. There are no observed interactions with the enzyme for this mannosyl unit.

The mannosyl group in the −3 subsite stacks hydrophobically with Tyr-317 but does not otherwise interact with the enzyme. A galactose branch is also possible in this subsite as the mannose unit O6 points out of the active-site cleft, without any clear protein interaction, with the closest residue being Tyr-317 about 4.5 Å away. In the −2 subsite, the mannosyl unit forms hydrogen bonds directly to Trp-314 and Tyr-315 and is part of hydrogen-bonding networks with Val-77, Asp-101, Glu-334, and His-335 (Table 4 and Fig. 5). The sugar unit O6 is involved in several of these interactions (Table 4), making galactose accommodation unlikely (Fig. 5).

Thus, G1M4 interacts strongly with *Bo*Man26B in the −5 subsite, the galactosyl side-group of the −4 subsite and the −2 subsite. Accommodation of galactose side-groups appears to be possible in all negative subsites except −2 and may even be favored in subsite −4. No other currently determined GH26 structure displays galactosyl side-group binding or accommodation in the −4 subsite, making the current structure of *Bo*Man26B in complex with G1M4 unique.

### Comparing BoMan26B with its two closest structural homologues

**Figure 6.**
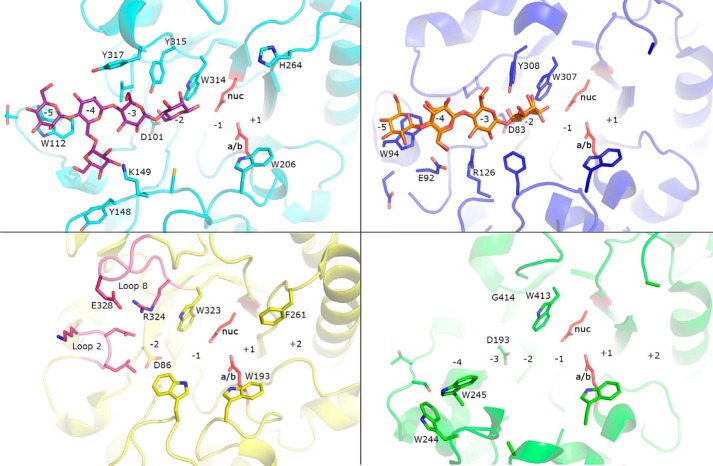
**Structural comparison of *Bo*Man26B (*light blue*), *Rs*Man26C (*dark blue*), *Pa*Man26A (*green*), and *Bo*Man26A (*yellow*).** The G1M4 saccharide bound in *Bo*Man26B is shown in a *stick* representation (*purple*) and the M4 is bound in *Rs*Man26C (*orange*). Loops 2 and 8 in *Bo*Man26A are colored *dark pink*. Subsite positions in each enzyme are *numbered.* The side chains of the catalytic acid/base (*a/b*) and nucleophile (*nuc*) are shown in *red* and labeled in *bold* for *Bo*Man26B (Glu-201 and Glu-291), *Rs*Man26C (Glu-191 and Glu-288), *Pa*Man26A (Glu-300 and Glu-390), and *Bo*Man26A (Glu-188 and Glu-292), respectively. In addition to the two catalytic residues, the following subsite −1 residues are conserved (*Bo*Man26B numbering): His-136, Arg-197, His-200, Phe-207, and Tyr-263 (side chains not shown) and Trp-314 (side chain shown). For *Bo*Man26B, side chains of residues that interact G1M4 are shown, and the side chains of the corresponding residues are shown for the other structures. The side chains of other residues discussed in the text are also shown. Amino acid numbers are according to each protein.

*Bo*Man26B was compared with the two closest structural homologues, *Podospora anserina* GH26 mannanase A (*Pa*Man26A) (39) and GH26 β-mannanase C from a gut symbiont of *Reticulitermes speratus* (*Rs*Man26C) (33), using a structural overlay (RMSD 0.75 Å and 0.82 Å for 207 and 182 Cα atoms, respectively). *Pa*Man26A has four negatively numbered, saccharide-interacting subsites, whereas *Rs*Man26C and *Bo*Man26B have five and structures containing glycans in subsites −5 to −2. The −5 subsite is similarly open in both *Bo*Man26B and *Rs*Man26C, with conserved hydrophobic stacking with Trp-112 (*Bo*Man26B numbering and Trp-94 in *Rs*Man26C, Fig. 6).

The −4 subsite has a low degree of conservation; *Bo*Man26B primarily interacts with the galactose side-group, whereas in *Rs*Man26C, the mannosyl moiety is part of a hydrogen-bonding network (33) and in *Pa*Man26A the substrate interacts strongly with this subsite, possibly due to Trp-244 and Trp-245 (39). Based on the current overlay, accommodation of a galactose side-group in the conformation observed in *Bo*Man26B is not possible for *Rs*Man26C, where Glu-92 and Arg-126 would clash, or *Pa*Man26A, in which Trp-244 and Trp-245 occupy the necessary space (Fig. 6).

In the −3 subsite of *Bo*Man26B and *Rs*Man26B, different residues are responsible for substrate interactions (Fig. 6). None of these residues are present in *Pa*Man26A, which has a weak −3 subsite (39). As for *Bo*Man26B, accommodation of a galactose side-group in subsite −3 is likely also for *Pa*Man26A and *Rs*Man26B (Fig. 6).

In the −2 subsites, Asp-101 and Trp-314, which in *Bo*Man26B are involved in substrate interaction, are conserved in all three structures. Ligand docking of *Pa*Man26A indicates that it is capable of galactose accommodation in the −2 subsite (28), something that would be blocked in *Bo*Man26B and *Rs*Man26C by Tyr-315 (*Bo*Man26B numbering), which is conserved in these two enzymes. In addition, the −2 subsite mannosyl unit O6 is partially responsible for substrate interaction with *Bo*Man26B and *Rs*Man26C (Fig. 6) (33).

The −1 subsite is generally conserved in GH26 β-mannanases (33, 44), including *Bo*Man26B, *Rs*Man26C, and *Pa*Man26A, and has been shown to be capable of harboring a galactose side-group pointing away from the active-site cleft in *Pa*Man26A and GH26 β-mannanase C from *Cellvibrio japonicus* (28, 32).

Thus, *Bo*Man26B, *Rs*Man26C, and *Pa*Man26A all have active-site clefts with long glycone-binding regions (*i.e.* several negative-numbered subsites) and relatively low degrees of conservation beyond subsite −1. The ability to accommodate galactose side-groups in these subsites varies: *Rs*Man26C appears to be restricted in two subsites (−4 and −2), whereas *Bo*Man26B and *Pa*Man26A only seem restricted in one subsite each, −2 and −4, respectively. Ligand-docking studies of *Pa*Man26A indicate that galactose could be accommodated in the +1 subsite (28), and based on comparisons with *Bo*Man26B and *Rs*Man26C, this seems likely for these two enzymes as well.

### Comparing BoMan26A and BoMan26B

**Figure 7.**
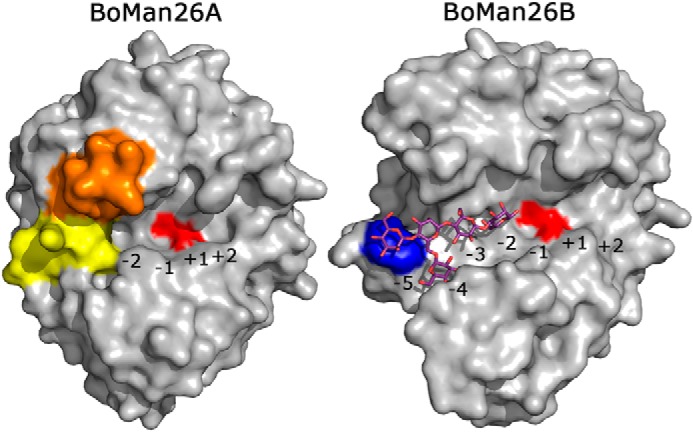
**Surface overviews of *Bo*Man26A (11) and *Bo*Man26B.** The G1M4 oligosaccharide bound in *Bo*Man26B is shown in *stick* representation. The subsites in each enzyme have been marked. The catalytic residues are shown in *red*; Trp-112 in *Bo*Man26B is shown in *blue*, and in *Bo*Man26A, the two significant loops (11) are colored: loop 2 (*yellow*) and loop 8 (*orange*).

A surface view of *Bo*Man26A (11) and *Bo*Man26B reveals a more open active-site cleft in *Bo*Man26B, with five subsites on the glycone side, whereas the *Bo*Man26A cleft is narrower and restricted by loops 2 and 8, resulting in only two glycone-binding subsites (Fig. 7). When superimposing *Bo*Man26B with *Bo*Man26A (11) (RMSD 2.20 Å for 149 Cα atoms), the loops around the active-site cleft show low conservation, except around the conserved catalytic residues, where mainly subsite −1 is invariable (Fig. 6) (33, 44).

The *Bo*Man26B aglycone side beyond the +1 subsite is wider than in *Bo*Man26A. Trp-206 in *Bo*Man26B is about 4 and 1.5 Å further away from backbones of helix α6 and the loop lining the bottom of the aglycone side compared with the corresponding Trp-193 in *Bo*Man26A (Fig. 6). In addition, *Bo*Man26A Phe-261 restricts the area above subsite +2 (8 Å to Trp-193) more than the equivalent His-264 in *Bo*Man26B (11 Å to Trp-206), which could limit the ability to accommodate galactose side-groups.

On the glycone side, beyond the −1 subsite, only two residues are conserved between *Bo*Man26A and *Bo*Man26B: Asp-101 and Trp-314 (Asp-86 and Trp-323 in *Bo*Man26A). Asp-101 is involved in subsite −2 substrate interaction (Fig. 5); Trp-314 generally provides hydrophobic stacking in the −1 subsite for GH26 β-mannanases (33, 44) and interacts directly with the bound mannosyl unit in the −2 subsite of *Bo*Man26B. Tyr-315 in *Bo*Man26B corresponds to Arg-324 in *Bo*Man26A, which forms a salt bridge in loop 8, possibly involved in restricting glycan binding (Fig. 6) (11). The other half of this salt bridge, Glu-328, corresponds to Tyr-317 in *Bo*Man26B, which provides stacking interactions in the −3 subsite. Another major difference is the position of loop 2. In *Bo*Man26A, loop 2 interacts with the −2 subsite mannosyl unit and blocks the −3 subsite (11). Loop 2 also interacts with loop 8, which may be flexible (54). The loop 2 equivalent in *Bo*Man26B is situated further away from the active site and includes Trp-112, essential for the −5 subsite (Fig. 6).

The longer and more open active-site cleft in *Bo*Man26B correlates with its preference for longer substrates and production of a range of oligosaccharide products (Fig. 2). In contrast, *Bo*Man26A has a shorter, narrower active-site cleft and preferentially produces M2 from manno-oligosaccharides (11).

### Mutational analysis of BoMan26B

Variants of *Bo*Man26B were generated to further study the preference for galactose at the −4 subsite (K149S and K149A) and the role of the −5 subsite in substrate binding (W112F and W112A). Resolved Michaelis-Menten kinetic constants (*k*_cat_ and *K_m_*) were obtained for the K149A and K149S variants on LBG, but not on guar gum or for the Trp-112 variants, as saturation could not be reached over the measurable concentration range due to the viscous nature of galactomannans. In these cases, *k*_cat_/*K_m_* was estimated at low substrate concentrations based on linear regression. For the Lys-149 variants, the *k*_cat_/*K_m_* was about 2.7-fold lower than *Bo*Man26B for both substrates (Table 1). For LBG, this was primarily due to a 2-fold increase in *K_m_*, with less effect on *k*_cat_ (decrease by a third), indicating that reduction of affinity was a main cause for the decrease in catalytic efficiency. The Trp-112 variants showed about 20-fold lower catalytic efficiency compared with the WT *Bo*Man26B (Table 1). With the prominent position of Trp-112 in the structure, it can be hypothesized that the loss of catalytic efficiency is due to reduced saccharide interaction at this position. To shed light on this issue, we performed a saccharide binding analysis with the Trp-112 variants.

**Figure 8.**
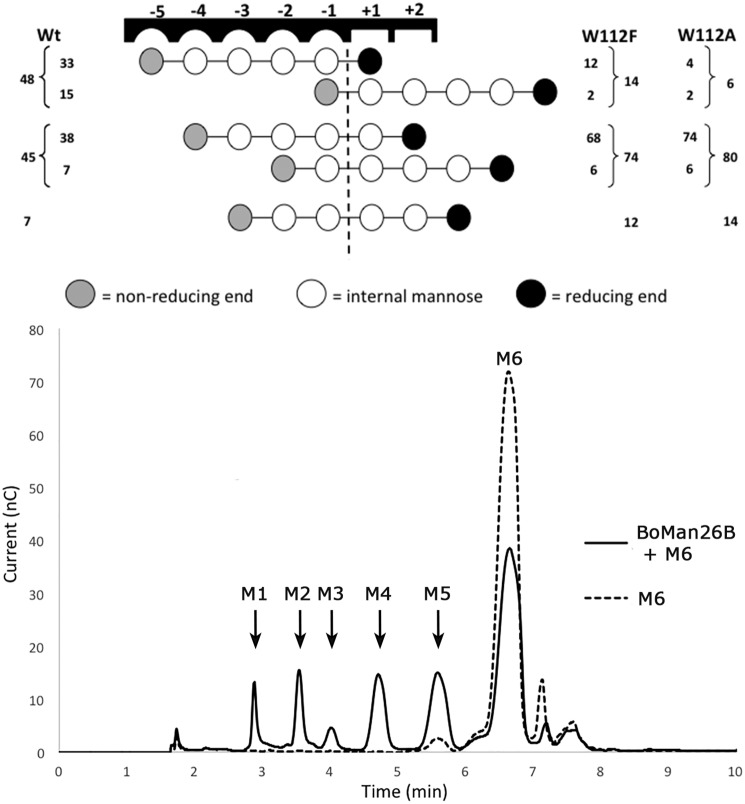
**Productive M6-binding modes for *Bo*Man26B (Wt) and two subsite −5 variants.** *Top:* ^18^O data showing the relative frequency (%) between different preferred productive binding modes of M6 for the Wt *Bo*Man26B and the variants W112A and W112F. Each *circle* represents a mannose unit with the *dashed line* between the −1 and +1 subsites showing the point of hydrolytic cleavage. HPAEC data were used to determine the relative frequency of oligosaccharides produced (outer frequency numbers), which was followed by determination of the ratios of ^18^O-labeled *versus* nonlabeled M5, M4, or M3 (MALDI-TOF MS) to determine the frequencies of modes giving the same products (inner frequency numbers). The mannose unit, which is present at the −1 subsite of each hydrolytic event, will be labeled with ^18^O upon incubation in ^18^O-labeled water. MALDI-TOF MS analysis thus can be used to determine the ratio of *e.g.* labeled M4 *versus* unlabeled, and these data were used to calculate the inner frequency numbers. *Bottom:* early to intermediate hydrolysis profile of *Bo*Man26B (Wt) on M6 is analyzed by HPAEC-PAD.

Thus, the mode of productive saccharide binding for *Bo*Man26B and variants W112F and W112A was determined to investigate the importance of Trp-112 in the −5 subsite. M6 hydrolysis in the presence of H_2_^18^O was analyzed by MALDI-TOF MS and HPAEC-PAD in accordance with previous studies (29, 39). The main hydrolytic events of *Bo*Man26B on M6 result in production of mannose (M1) and M5, as well as M2 and M4 (Fig. 8). The variants W112F and W112A primarily increased the production of M2 and M4, at the expense of M1 and M5 production (Fig. 8). The two dominant productive M6-binding modes of *Bo*Man26B are from subsite −5 to +1 and −4 to +2, which together represent more than two-thirds of all hydrolysis events (Fig. 8). W112F and W112A shift the dominant productive M6-binding mode to spanning subsites −4 to +2, with −5 to +1 binding being strongly suppressed (Fig. 8). This highlights the importance of Trp-112 in creating a strongly binding −5 subsite and reveals a clear preference for saccharide binding in the glycone over the aglycone subsites, similar to other GH26 enzymes (36, 39).

### Phylogenetic analysis of GH26

**Figure 9.**
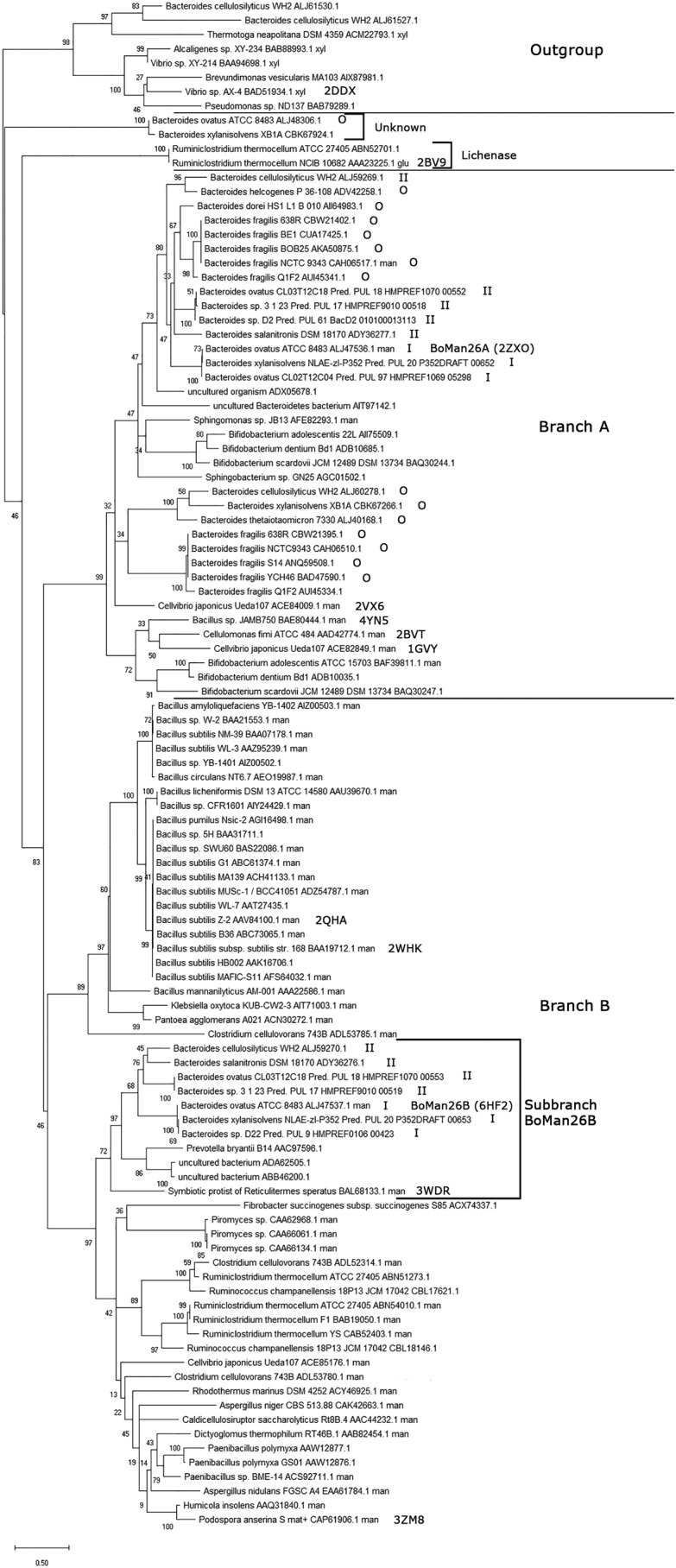
**Phylogenetic tree of selected GH26 sequences.** *Bo*Man26A and *Bo*Man26B are labeled, as well as those sequences with a determined structure (showing their PDB codes) and the sequences that are encoded by type I, type II, or other (*O*) PULs. The outgroup contains all sequences of enzymes with known xylanase activity. The two major branches with β-mannanases are labeled *branch A* and *B*. Sub-branch *Bo*Man26B, used for a subsequent multiple sequence alignment (Fig. S5), is also marked. Two other major branches are only represented by two sequences each and have unknown or lichenase activity, respectively.

Several other *Bacteroides* strains, which are capable of growing on galactomannans, have predicted homologous PULs to *Bo*ManPUL, all including two putative adjacent GH26 β-mannanase genes (Fig. 1*A*) (43). The GH26 β-mannanases encoded by these genes are here termed “GH26 pairs.” The compared homologous PULs were split into two types based on whether they contained a putative GH36 α-galactosidase gene (type I) or not (type II, Fig. 1) (43). To study the difference between types I and II β-mannanases on a sequence level and place the “GH26 pair” *Bo*Man26A and *Bo*Man26B in context of other GH26 enzymes, a phylogenetic tree was generated. This tree was composed of characterized GH26 enzymes and *Bacteroides* GH26 enzymes with entries in CAZy, as well as putative “GH26 pairs” from the type I and type II PULs listed previously (43). The sequences clustered into five major branches (Fig. 9). Apart from the outgroup (GH26 xylanases), there were two branches containing only two sequences each (with unknown enzymes/lichenases) and two large branches (A and B), which included all characterized β-mannanases (Fig. 9). The branch with unknown function includes a putative GH26 enzyme that was previously predicted to be a β-mannanase (GenBank^TM^ accession no. ALJ48306.1) (43), which thus may display another specificity.

All characterized β-mannanases and all GH26 pairs encoded by type I and type II PULs clustered into branches A and B. The two enzymes of each GH26 pair clustered in different branches, as exemplified by *Bo*Man26A (branch A) and *Bo*Man26B (branch B, Fig. 9). All GH26 pair enzymes similar to *Bo*Man26B clustered together in the *Bo*Man26B sub-branch, which also encompassed four additional sequences, including *Rs*Man26C (Fig. 9).

*Bacteroides* GH26 enzymes from other PULs (the majority with only one GH26 enzyme) clustered in branch A. Enzymes in branch B come from more varied organisms, although one part is dominated by a number of highly similar *Bacillus* sequences (Fig. 9). This phylogenetic tree shows that *Bo*Man26A and *Bo*Man26B and similar GH26 pairs are relatively distantly related in evolution and thus that a GH26 pair may have been present in a parental PUL rather than that gene duplication has occurred several times within homologous PULs.

### Bioinformatic comparison of BoMan26B with other GH26 β-mannanases

The multiple sequence alignment used to generate the phylogenetic tree revealed 21 highly-conserved residues (≥97% conservation) in branches A and B, out of which four were strictly conserved (100%) in all compared GH26 sequences (Table 5). These 21 residues included seven residues previously reported to be conserved in GH26 β-mannanases (Table 5) (33, 44). Based on the *Bo*Man26B structure, highly conserved residues located in the active-site cleft were primarily found in subsites −1 and +1. His-200 is part of the HE motif thought of as a defining feature of GH26 enzymes (44) but is not conserved in the xylanase branch or in the branch with unknown function. Worth noting is that Trp-314 is highly conserved in the sequence alignment (Table 5) but is not structurally conserved between branches.

**Table 5:**
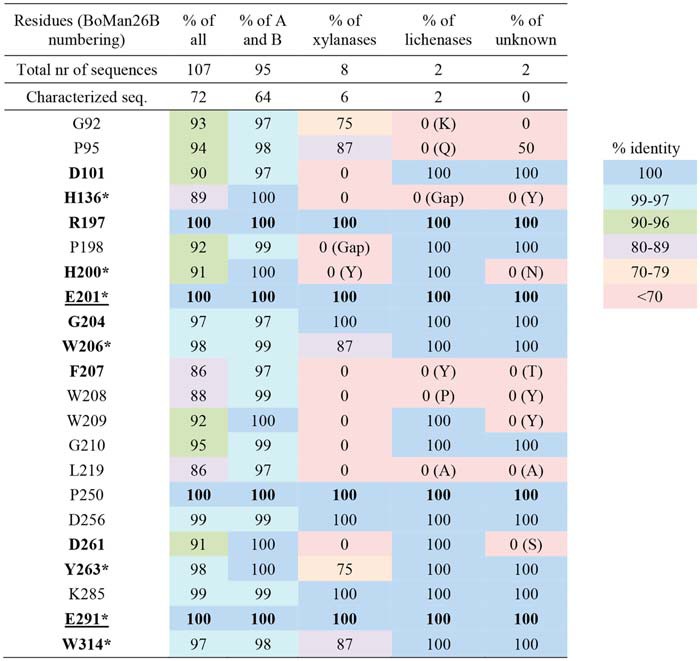
**Overview of conserved GH26 residues in all sequences from the phylogenetic tree** Residues with a minimum of 97% conservation in branches A and B are shown. Characterized sequences are based on the CAZy database (www.cazy.org).^4^ Conservation within sub-branches other than A and B is shown in parentheses. Residues in the active-site cleft are marked in bold, with the catalytic residues (E201 and E291) underlined (50). These residues are in the following subsites D101 (−2), H136 (−1), R197 (−1), H200 (−1), G204 (+1), W206 (+1), F207 (−1), D261 (+1), Y263, (−1) and W314 (−1).

* Residues were previously stated to be conserved in GH26 β-mannanases (33, 44).

*Bo*Man26B was compared with the other sequences in its sub-branch and the GH26 pair sequences from branch A using a multiple sequence alignment. All highly conserved residues in branches A and B (Table 5) aligned in the included sequences (Fig. S5). There is an overall conservation of residues around subsite −1 and +1 (Table 5) but variations in other subsites. For example, Trp-112 in the −5 subsite and Tyr-315 that restricts galactose in the −2 subsite in *Bo*Man26B (Fig. 6) are not present in branch A and are only conserved in sub-branch *Bo*Man26B (Fig. S5). Tyr-148 and Lys-149, which interact with the galactose side-group at subsite −4 in *Bo*Man26B (Fig. 6), are only conserved in *Bo*Man26B-like enzymes from GH26 pairs encoded by type I PULs (Fig. S5).

Thus, the relatively high levels of conservation around the −1 and +1 subsites, but otherwise low levels of conservation around the active-site cleft, could imply that even rather closely related mannanases may differ in the detailed fine-tuned substrate specificity. Prediction may be facilitated if more sequences of characterized enzymes would be added to a phylogenetic analysis.

## Discussion

### Importance of BoManPUL

To enable routes for better health through diet, it is imperative to understand how our gut microbiota utilizes dietary fibers (5). β-Mannans are a part of our diet (19, 20), but there have been relatively few studies carried out on their utilization by human gut bacteria (11, 30, 40, 41). *B. ovatus* is a common human gut bacterium with several PULs for hemicellulose utilization, including the previously identified *Bo*ManPUL with two GH26 β-mannanases, *Bo*Man26A and *Bo*Man26B (16). Characterization of *Bo*Man26A and partial characterization of *Bo*Man26B revealed that the activity of *Bo*Man26A is restricted by the galactosyl decorations in galactomannans, whereas that of *Bo*Man26B is not (11). These two mannanases also differ in their generated products: *Bo*Man26A produces primarily M2 from β-mannan substrates, whereas *Bo*Man26B is a more random endo-acting enzyme because it produces a range of oligosaccharide products (Fig. 2) (11). The current study of *Bo*Man26B further relates the biochemical data on substrate and product preferences to the determined crystal structure and further defines the role of *Bo*Man26B in the *Bo*ManPUL-encoded system for galactomannan utilization. *Bo*Man26B is placed in context of other members of the GH26 family, expanding the current view on GH26 and putative galactomannan-related PULs.

### Role of BoMan26B in BoManPUL

The previously proposed model of galactomannan utilization by the proteins encoded by *Bo*ManPUL is supported by the current data, and new insight is gained. According to the model, the extracellular *Bo*Man26B is the enzyme performing initial galactomannan attack, followed by the periplasmic *Bo*Gal36A and *Bo*Man26A (Fig. 1*B*) (11). In this study, the decrease in *k*_cat_/*K_m_* on guar gum compared with LBG for *Bo*Man26B further confirms the low levels of restriction by galactose side-groups for this enzyme (Table 1). *Bo*Man26B produces manno-oligosaccharides, many of which are decorated with galactosyl units (Fig. 2). The galactomannan breakdown to oligosaccharides facilitates galactosyl removal by *Bo*Gal36A as indicated by the sequential synergy observed for *Bo*Man26B and *Bo*Gal36A (Table 2). *Bo*Gal36A has previously been shown to aid the action of *Bo*Man26A, which is severely restricted by galactose decorations (11). Although synergistic action of GH enzymes from the same gut bacterial PUL has previously been shown for PULs involved in xylan utilization (55, 56), this is the first time such synergy has been shown for all GH enzymes in a gut bacterial PUL involved in β-mannan utilization.

The new data on the *Bo*ManPUL-encoded SusD-like protein fits into the model, because it has an assumed outer-membrane function in connection to the predicted SusC-like transporter and binds galactosylated and linear manno-oligosaccharides (Fig. 3) of a similar DP as those produced by *Bo*Man26B (Fig. 2). The SusD-like protein can also bind galactomannan, but the affinity was not quantified (11). Other SusD-like proteins are known to bind shorter glycans (57) or polysaccharides (58). A few studies on starch utilization have shown that SusD functions in complex with the outer membrane transporter SusC (59, 60). Thus, although we can confirm the role of the SusD-like protein to bind mannose-based glycans, it would be interesting to further investigate any synergistic effects with other *Bo*ManPUL proteins.

Thus, the previously proposed model of galactomannan utilization encoded by the *Bo*ManPUL (11) is further confirmed by the current study on *Bo*Man26B and the SusD-like glycan binding protein. This system has general similarities but also specific differences, compared with other *B. ovatus* PULs (16) involved in utilization of other hemicellulosic polysaccharides. The more complex structure of xylan and xyloglucan is reflected by corresponding *B. ovatus* PULs encoding larger numbers of extracellular and periplasmic GHs (61, 62). The GHs in the current study lack carbohydrate-binding modules. This is often the case for PUL-encoded GHs, with one of the known exceptions found for a GH involved in xylan-utilization (61).

### G1M4 binding to BoMan26B

The G1M4 complex structure and mutational studies reveal strongly interacting −5 and −2 subsites in *Bo*Man26B, with a suggested preference for a galactosyl side-group at the −4 subsite, where several enzyme–galactosyl interactions occur (Fig. 5). Previously, galactosyl side-group interactions with other crystallized GH26 mannanase have been observed for either subsite −1 or −2 (32, 63), but not for subsite −4. Some of the *Bo*Man26B residues important for mannan and galactosyl side-group binding are seen in the structure. The main subsites responsible for binding the mannosyl units of G1M4 are −5 and −2, with limited or no interaction at the −4 and −3 subsites (Fig. 5). The importance of Trp-112 for a strong −5 subsite, which likely is a contributing factor to the preference of *Bo*Man26B for longer substrates, was confirmed by the binding mode and kinetics of the W112F and W112A variants (Fig. 8 and Table 1). Differences in size of the aromatic rings and electrostatic potentials between tryptophan and phenylalanine (64) cause the large shift in sugar stacking ability between these two residues. Trp-112 is situated in loop 2 that is also involved in the calcium-binding site (Fig. S4). Thus, the calcium-binding site may play an important role in the correct conformation of the −5 subsite.

The possible preference for a galactosyl side-group at the −4 subsite is seen in the G1M4-complex structure, as Tyr-148 and Lys-149 hydrogen bonds with the galactose side-group. Positive interaction with the galactosyl side-group is further indicated by the analysis of the Lys-149 variants, which likely remove one of the interactions, which explains the increase of *K_m_* and the decrease in catalytic efficiency using galactomannan substrates (Table 1).

Despite the enzyme's low levels of restriction by galactose decorations, no hydrolysis of G2M5 by *Bo*Man26B was detected. The preference for substrate binding in subsite −5, possible interaction with a side-group in the −4 subsite, and a restriction toward galactose side-groups in the −2 subsite may explain the enzyme's inability to hydrolyze G2M5, as it would bind in subsites −5 to −1.

### Comparing the BoMan26B active-site cleft with BoMan26A, RsMan26C, and PaMan26A

The biochemical differences observed between *Bo*Man26B, *Bo*Man26A, *Rs*Man26C and *Pa*Man26A relates to their structures. *Bo*Man26B (Fig. 7) and *Bo*Man26A (11) differ in the overall shape of their active-site clefts, which is reflected in their mode of attack. *Bo*Man26A has a narrow active-site cleft with only two glycone subsites, restricted by loop 2, and mainly produces M2, indicative of exo-activity with some endo-acting capability (11). In contrast, *Bo*Man26B is endo-acting because it prefers longer substrates and produces a variety of oligosaccharide products from galactomannan (Fig. 2), as explained by a wider active-site cleft, where a shorter loop 2 harbors Trp-112 crucial for the −5 subsite (Fig. 7).

*Bo*Man26B and *Pa*Man26A (28) are suggested to be limited in their ability to accommodate galactose side-groups in only one glycone subsite, whereas both *Rs*Man26C (31) and *Bo*Man26A (11) are restricted in at least two subsites. This is reflected in their catalytic efficiency or specific activity on guar gum compared with LBG, which is reduced to a much greater degree for *Bo*Man26A (11) and *Rs*Man26C (31) than for *Bo*Man26B (Table 1) and *Pa*Man26A (28).

Thus, clear differences in the active-site clefts of the four enzymes *Bo*Man26B, *Bo*Man26A, *Pa*Man26A, and *Rs*Man26C correlate with differences in activity and product profiles. A longer active-site cleft increases the variation in products produced, and a greater restriction by galactose side-groups is reflected by a larger number of subsites unable to accommodate them.

### BoMan26B in context of other GH26 enzymes

The phylogenetic tree and bioinformatic analysis of *Bo*Man26B and related enzymes shed light on differences and similarities in GH26. Only two of the five major branches in the tree contain β-mannanases, whereas the others harbor xylanases, lichenases, and one branch of unknown function (Fig. 9). It would be interesting to incorporate more sequences in the tree as these five branches may hint at a possibility for subfamily classification of GH26, *e.g.* as has been done for the large GH5 family that also harbors mannanases and several other specificities (65). Several GH26 β-mannanases with 2–3 known negative subsites, including *Bo*Man26A, cluster in branch A (GenBank^TM^ accession numbers ACE84009.1, ADD42774.1, and ACE82849.1) (32, 36, 44), whereas all GH26 β-mannanases known to have four or five negative subsites with a determined structure are found in branch B (GenBank^TM^ accession numbers BAL68133.1, AAV84100.1, and CAP61906.1) (33, 35, 39). The β-mannanases with differing active-site cleft structures cluster in different branches, which may point to an overall difference between branches A and B.

The highly conserved residues in branches A and B were mainly located in the −1 and +1 subsites. Each enzyme in the compared “GH26 pair” sequences, including *Bo*Man26A and *Bo*Man26B, clusters into two separate phylogenetic groups (Fig. 9) suggesting that each member of such a pair has a distinct fine-tuned function that cannot be replaced by the other, which is also supported by the observed large differences in active-site structure, product profile, and substrate preference.

The conservation of Trp-112 and Tyr-315 in all sub-branch *Bo*Man26B sequences points toward common features in the active-site cleft: the presence of a −5 subsite and restriction of galactose side-group accommodation in the −2 subsite (Fig. S5).

Thus, two distinct branches of β-mannanases are seen in the phylogenetic tree, where the enzymes present in branch B possibly have longer active-site clefts than in branch A. Four residues are strictly conserved cross-GH26 sequences, but several residues mainly located around subsites −1 and +1 are in addition highly conserved in GH26 mannanases (branches A and B). Variation is larger in other subsites, and the residues involved in galactose accommodation in *Bo*Man26B are only conserved in a small part of branch B.

## Conclusion

This study shows that *Bo*Man26B has low levels of restriction by galactose side-groups, which can be accommodated in glycone subsites −5, −4, −3, and −1, and generates a range of manno-oligosaccharide products of DP 2–5 from galactomannan. Our new data on *Bo*Man26B and *Bo*Gal36A synergy, and the glycan binding of the SusD-like protein, further emphasize our model (11) for galactomannan degradation by *B. ovatus*, conferred by *Bo*ManPUL. Outer membrane attached *Bo*Man26B initiates galactomannan hydrolysis and produces galactomanno-oligosaccharides. These are bound to the SusD-like protein and transported into the periplasm where *Bo*Gal36A releases the galactose units, followed by *Bo*Man26A-catalyzed hydrolysis of linear manno-oligosaccharides into M2, which is internalized. The prominent −5 subsite in *Bo*Man26B likely contributes to a preference for substrates longer than DP5. The ability to accommodate galactose is less restricted for *Bo*Man26B than for several other GH26 enzymes, which have heavily reduced activity on guar gum compared with LBG. *Bo*Man26B clusters in a different major phylogenetic branch of β-mannanases than *Bo*Man26A, reflected in their significant differences in structure and biochemistry. The region by the −4 subsite in *Bo*Man26B, which is responsible for galactose coordination, is not conserved, except within the type I *Bo*Man26B-like enzymes. These data further place *Bo*Man26B in context of other GH26 enzymes and reveal the structural basis for detailed substrate specificity of this enzyme.

## Experimental procedures

### Materials

M2–M6, G2M5, GM3, GM2, low-viscosity LBG, medium-viscosity guar gum galactomannan, and guar α-galactosidase were from Megazyme (Bray, Ireland). LBG was from Sigma; M1 was from Fluka (Steinheim, Germany); potassium phosphate buffer, imidazole, CaCl_2_, and NaH_2_PO were from Merck (Darmstadt, Germany). All other chemicals, unless stated otherwise, were from Sigma. LBG and low-viscosity LBG have a galactosyl unit/mannosyl unit ratio of 1:4, whereas for guar gum and medium-viscosity guar gum this ratio is 1:2. Low-viscosity LBG and medium-viscosity guar gum were used for the kinetics.

### BoMan26B expression and purification

*Bo*Man26B was expressed as described previously (11) and purified with slight modifications: *Escherichia coli* BL21(DE3) cells containing expressed protein were dissolved in lysis buffer (20 mm NaH_2_PO_4_, 0.5 m NaCl, 1 mm CaCl_2_, and 20 mm imidazole, pH 7.4) and lysed by a French press. After subsequent centrifugation (JA 25.50 rotor, 21,500 rpm, 4 °C, 30 min), a HisTrap^TM^ HP 1-ml column (GE Healthcare, Pollards Wood, UK) was equilibrated with lysis buffer on a BioLogic DuoFlow chromatography system (Bio-Rad) at 10 °C. The sample was loaded on the column that was washed with 20 ml of lysis buffer, followed by a gradient of 0–100% elution buffer (lysis buffer, with 400 mm imidazole) over 20 ml, collecting 1-ml fractions at 1 ml/min. The purity of the fractions was assessed with SDS-PAGE (Fig. S6), and relevant fractions were pooled, and the buffer was changed to 50 mm MES buffer, pH 6.5, with 1 mm CaCl_2_ (storage buffer) (11).

### Variants of BoMan26B

*Bo*Man26B synthetic gene variants (inserted in the BoMan26B expression plasmid) were purchased from GenScript (Piscataway, NJ) (Table S1) and transformed into One Shot^TM^ BL21(DE3) chemically competent *E. coli* (Invitrogen, Thermo Fisher Scientific), and protein was expressed and purified as *Bo*Man26B. The coding regions was sequenced using T7 primers (Eurofins Genomics, Edersberg, Germany).

### β-Mannanase activity assay, calcium stability, and kinetics

Activity was measured using the standard 3,5-dinitrosalicylic acid (DNS)-reducing sugar assay using 0.5% (w/v) LBG in 50 mm potassium phosphate buffer, pH 6.5, and 0.7 μg/ml (18 nm) *Bo*Man26B for 15 min at 37 °C as described previously (11, 66). A mannose standard curve was used. The specific activity of *Bo*Man26B with LBG was 99.7 ± 9.2 katal/mol. Metal stability was assessed by incubating *Bo*Man26B at 37 °C in the presence of 1 mm EGTA or CaCl_2_ in 50 mm MES buffer, pH 6.5. Aliquots were removed at 0, 1, 3, and 24 h and stored at 4 °C, and their activity was measured simultaneously. To determine the effect of calcium on activity, 1 mm CaCl_2_ or EGTA was included in the assay.

Michaelis-Menten kinetics was measured for 50 ng/ml *Bo*Man26B and the variants K149S, K149A, W112F, and W112A in triplicate using the DNS activity assay but varying time and substrate concentration. All reactions had a total volume of 0.4 ml and contained 1 mm CaCl_2_ and LBG (20, 17.5, 15, 12.5, 10, 7.5, 5, 4, 3, and 2.5 g/liter for *Bo*Man26B; 20, 15, 12.5, 10, 7.5, 5, and 3 g/liter for the Lys-149 variants; and 20, 15, 10, 7.5, 5, and 3 g/liter for the Trp-112 variants) or medium-viscosity guar gum (15, 12.5, 10, 7.5, 5, 4, 3, and 2.5 g/liter for *Bo*Man26B, and 16.5, 15, 12.5, 10, 7.5, 5, and 3 g/liter for the Lys-149 variants, and 16.5, 15, 12.5, 10, 7.5, and 5 g/liter for the Trp-112 variants). Aliquots were removed at three time points, and the reaction was stopped by adding DNS. The obtained initial rates were used to generate Michaelis-Menten curves using GraphPad Prism 7.04 (La Jolla, CA) from which *K_m_* and *k*_cat_ values were determined. Because of the viscosity of the substrates, for some of the variants, initial rates could not be obtained at sufficiently high substrate concentration (Fig. S1). So, in addition to obtaining resolved *K_m_* and *k*_cat_ values, the linear slope of *V*_0_ *versus* low substrate concentrations was used to estimate *V*_max_/*K_m_*, and thereby *k*_cat_/*K_m_*, as described previously (29). For *Bo*Man26B and the Lys-149 variants on low-viscosity LBG, the estimated *k*_cat_/*K_m_* was similar to the values obtained when fitting the data to classic Michaelis-Menten kinetics (Table 1), indicating a reliable approach. For the linear regression the following substrate concentrations were used: 3, 5, and 7.5 g/liter for K149A and K149S and 5, 7.5, and 10 g/liter for W112F and W112A.

### Product length generated by BoMan26B

5.5 μm *Bo*Man26B was incubated with 0.5% (w/v) LBG or guar gum and 1 mm CaCl_2_ in 50 mm potassium phosphate buffer, pH 6.5, at 37 °C in duplicate for 24 h before boiling for 10 min. 50 μl of the reaction mixture was re-equilibrated to 37 °C before adding 0.5 units of guar α-galactosidase (Megazyme, Bray, Ireland) (1 unit is the amount of enzyme required to release 1 μmol of product/min), incubated for another 24 h at 37 °C, before boiling for 10 min. The samples containing hydrolysis products with or without treatment with guar α-galactosidase were analyzed by HPAEC-PAD with CarboPac PA-200 and PA-20 columns (Dionex, Sunnyvale).

### Synergy of BoMan26B and BoGal36A

Synergy between *Bo*Man26B and *Bo*Gal36A was analyzed with LBG and guar gum as described previously for *Bo*Man26A and *Bo*Gal36A (11), using 1.4 μm of each enzyme. The amount of M2 and galactose generated from the incubations was quantified by HPAEC-PAD to determine differences in product release when LBG and guar gum were incubated with only one enzyme or both.

### Purification of the SusD-like protein

The gene construct for the SusD-like protein, generated previously (11), was transformed into One-shot^TM^ BL21(DE3) chemically competent *E. coli* (Invitrogen, Thermo Fisher Scientific). The protein was expressed and purified as described previously (11) but using French press for cell lysis.

### Sugar-binding studies of SusD

The affinity toward G2M5, M6, and M5 of the SusD-like protein was examined using MST. The cysteines of the SusD-like protein were labeled with the Monolith protein-labeling kit Red-maleimide (NanoTemper, Munich, Germany) in 20 mm HEPES, 0.1 m NaCl, pH 7.5. 100 μl (20 μm) of protein was mixed with 100 μl (60 μm) of dye and incubated for 1 h in the dark at room temperature. Column B of the labeling kit was equilibrated with analysis buffer (20 mm HEPES, 0.1 m NaCl, and 0.05% Tween 20, pH 7.5) before loading the incubated sample, washing with 300 μl of analysis buffer, and eluting the sample in three 200-μl fractions using the analysis buffer. Nanodrop was used to determine protein concentration using the molar extinction coefficient 138,465 m^−1^ cm^−1^ and the molecular mass of 65.6 kDa (ProtParam ExPASy server) (67).

200 mm G2M5, M6, or M5 in analysis buffer was diluted in analysis buffer in a sequential 1:1 dilution series to a final volume of 10 μl. 10 μl of 200 nm SusD-like protein in analysis buffer was added to each tube for a final concentration of 100 nm SusD-like protein and a concentration range of 100 mm to 3.05 μm oligosaccharide. The reaction mixtures were incubated for 5 min at room temperature, loaded into premium capillaries, and analyzed with a Monolith NT.115 (NanoTemper, Munich, Germany) in duplicate at 37 °C using an excitation power of 50% and MST power of 20, 40, and 60%. A control SD-test was carried out with the SusD-like protein boiled with SDS and DTT. The control protein *Tm*NrdD (labeled with the same fluorophore) was incubated with G2M5 over the same concentration range as above. Data analysis was performed using the software MO.Affinity Analysis v2.3 (NanoTemper).

### Crystallization and data collection of BoMan26B

A pre-crystallization test (Hampton Research, Aliso Viejo, CA) was used to determine the optimal protein concentration for crystallization of *Bo*Man26B, assessing 7.4, 3.7, 1.9, 0.37, and 0.19 mg/ml. Based on these results, vapor diffusion (sitting drop) PACT and JCSG+ commercial screens (Molecular Dimensions, Newmarket, UK) were set up using a mosquito pipetting robot (TTP Labtech, Melbourne, UK) with drop sizes of 100 nl reservoir and 100 nl 5 or 2.5 mg/ml *Bo*Man26B in 50 mm MES, pH 6.5, with 0.6 mm CaCl_2_. The plates were stored at 20 °C in a Gallery 500 plate hotel (Rigaku, Sevenoaks, UK). Further screens were set up using 5 mg/ml *Bo*Man26B in 50 mm MES, pH 6.5, with 0.6 mm CaCl_2_ with hanging drop, and stored at 20 °C. Two crystals grown under the following conditions were used for data collection: 0.1 m bis-tris, 20% (w/v) PEG, 0.2 m NaCl. One crystal (apoenzyme) was grown at pH 4.8 using PEG4000. BoMan26B did not cleave G2M5 in incubations analyzed with HPAEC as described previously (11). For the second crystal, G2M5 (10 mm) was included at pH 5.0 using PEG6000 and 50 mm CaCl_2_. Glycan soaking of this crystal was carried out for the G1M4 complex structure in a drop containing 50% reservoir solution with 25 mm MES buffer, pH 6.5, 30% PEG400, and 25 mm G2M5, GM3, and GM2/M3 (Megazyme, Bray, Ireland) for 48 h.

Data collection was carried out at 100 K at the P13 beamline at PETRA III (DESY, Hamburg, Germany) with an X-ray wavelength of 1.0332 Å (apoenzyme) or at the ID29 beamline at the ESRF (Grenoble, France) at 0.96862 Å (G1M4 complex). The crystals were soaked in cryoprotectant (50% reservoir solution, 25 mm MES buffer, pH 6.5, with 30% PEG400) and flash-cooled in liquid nitrogen. Generation of an MTZ file, including indexing, integration of diffraction images, and scaling of the data were carried out with the XDS program suite (68) and CAD from CCP4 (69). A suitable model for molecular replacement for the apoenzyme structure was found using MrBUMP from CCP4 (69–71). Molecular replacement was carried out with *Pa*Man26A (PDB code 3ZM8, sequence identity 37% (39)) for the apoenzyme structure, subsequently used for the G1M4 structure using the Phenix version of Phaser-MR (71, 72), followed by several cycles of restrained refinement in Phenix (72) and manual editing in Coot (73). Of the 36 N-terminal residues not visible in the crystal structures, the first 19 residues constitute a signal peptide not part of the crystallized construct, whereas the remaining 17 residues are not visible. The G1M4 saccharide was modeled with sugar units in the ^4^C_1_-chair conformation. For structural comparison, the G1M4 complex structure was superposed to the two closest structural homologues of *Bo*Man26B: *Pa*Man26A, *Rs*Man26C (PDB code 3WDR, 34% sequence identity to *Bo*Man26B (33)) and *Bo*Man26A, using PyMOL (49).

### ^18^O labeling

The preferred productive binding mode of M6 to *Bo*Man26B and its variants was studied by performing hydrolysis in ^18^O-water, followed by analysis with MALDI-TOF MS and HPAEC-PAD according to the method described by Hekmat *et al.* (29). All samples were prepared in duplicate, incubated for 1 h (*Bo*Man26B) or 3 h (variants W112A and W112F), and run in parallel at 8 °C to avoid spontaneous ^18^O-labeling of saccharides. Incubations were performed using 2 mm M6, 1 mm CaCl_2_, and 5 μm (*Bo*Man26B) or 10 μm (W112A or W112F) enzyme in 0.6 mm potassium phosphate buffer, pH 6.5, with MilliQ water as solvent for HPAEC-PAD analysis and 97% H_2_^18^O, with a final H_2_^18^O content of 90% (*Bo*Man26B), 92% (W112F), and 93% (W112A) for the MALDI-TOF analysis. The reactions for HPAEC-PAD were stopped by adding a 20-μl sample to 980 μl of boiling water, and products were quantified using HPAEC-PAD with a CarboPac PA-200 column. The MALDI-TOF reactions were stopped by adding 0.5 μl of sample onto 0.5 μl of matrix (10 mg/ml 2,5-dihydroxybenzoic acid in 10 mm Na^+^) on a stainless-steel plate that was immediately dried with warm air. The samples were analyzed with a 4700 Proteomics Analyzer (Applied Biosystems, Foster City, CA) and processed as described previously (11, 29). The relative frequencies of the productive binding modes obtained from the MALDI-data were calculated based on M5 and M4.

### Phylogenetic analysis of GH26

The catalytic modules of the following 107 protein sequences were selected for generation of a phylogenetic tree: all characterized GH26 enzymes and all *Bacteroides* GH26 enzymes in the CAZy database (www.cazy.org),^4^ as well as GH26 pair sequences encoded by *B. ovatus, Bacteroides xylanisolvens,* and *Bacteroides* species D22, *Bacteroides* species D2, and *Bacteroides* species 3_1_23 PULs previously identified as being homologous to *Bo*ManPUL (43). A few GH26 sequences encoded by PULs were identical, and in these cases only one of them was used. The evolutionary history was inferred by using the Maximum Likelihood method based on the JTT matrix-based model (74), selecting the tree with the highest log likelihood (−13193.86). Initial tree(s) for the heuristic search were obtained automatically by applying Neighbor-Join and BioNJ algorithms to a matrix of pairwise distances estimated using a JTT model, and then selecting the topology with superior log likelihood value. A discrete γ distribution was used to model evolutionary rate differences among sites (five categories (+G, parameter = 1.6526)). The rate variation model allowed for some sites to be evolutionarily invariable ([+I], 1.61% sites). The tree was drawn to scale, with branch lengths measured in the number of substitutions per site. All positions containing gaps were eliminated, and there were 124 such positions in the final dataset. Evolutionary analyses were conducted in MEGA X (75).

### Bioinformatics of BoMan26A and BoMan26B PUL pairs

The multiple sequence alignment generated for the phylogenetic tree was used for analysis of conserved residues, both within the tree as a whole as well as within the different branches, with a focus on *Bo*Man26B.

A multiple sequence alignment containing the GH26 pairs and four additional sequences in sub-branch *Bo*Man26B of the phylogenetic tree (Fig. 9, GenBank^TM^ accession numbers AAC97596.1, ADA62505.1, ABB46200.1, and BAL68133.1) was generated using Clustal (https://www.ebi.ac.uk/Tools/msa/clustalo/)^4^ (77). The multiple sequence alignment was correlated with the active-site cleft structures of *Bo*Man26B and *Rs*Man26C (33).

## Author contributions

V.B. and H.S. conceptualization; V.B., D.T.L., and H.S. data curation; V.B., D.T.L., and H.S. formal analysis; V.B., A.R., D.T.L., and H.S. supervision; V.B., D.T.L., and H.S. validation; V.B., M.W., S.K.R., and A.B. investigation; V.B., S.K.R., and M.W. visualization; V.B., M.W., S.K.R., and A.B. methodology; V.B. writing-original draft; V.B., M.W., S.K.R., A.B., A.R., D.T.L., and H.S. writing-review and editing; H.S. funding acquisition; H.S. project administration.

## Supplementary Material

Supporting Information

## Footnotes

4 Please note that the JBC is not responsible for the long-term archiving and maintenance of this site or any other third party hosted site.

## Footnotes

3 The abbreviations used are:

GH: glycoside hydrolase
*Bo*Gal36A: GH36 α-galactosidase from *B. ovatus*
*Bo*Man26A and -B: GH26 β-mannanase A and B from *B. ovatus*
*Bo*ManPUL: polysaccharide utilization locus *bacova_02087-97* from *B. ovatus*
CAZy: carbohydrate-active enzyme
DNS: 3,5-dinitrosalicylic acid
DP: degree of polymerization
G1M4: galactosyl-mannotetraose
G2M5: digalactosyl-mannopentaose
GM2: 6^1^-α-d-galactosyl-mannobiose
GM3: 6^1^-α-d-galactosyl-mannotriose
HPAEC-PAD: high-performance anion-exchange chromatography with pulsed amperometric detection
HTCS: hybrid two-component system
LBG: locust bean gum
M1: mannose
M2: mannobiose
M3: mannotriose
M4: mannotetraose
M5: mannopentaose
M6: mannohexaose
MST: microscale thermophoresis
*Pa*Man26A: GH26 β-mannanase from *P. anserina*
PUL: polysaccharide utilization locus
RMSD: root mean square deviation
*Rs*Man26C: GH26 β-mannanase C from a gut symbiont of *R. speratus*
Sus: starch-utilization system
*Tm*NrdD: ribonucleotide reductase from *T. maritima*
bis-tris: 2-[bis(2-hydroxyethyl)amino]-2-(hydroxymethyl)propane-1,3-diol
PDB: Protein Data Bank
S-test: SDS denaturation test.

## References

[1] Kim S., Goel R., Kumar A., Qi Y., Lobaton G., Hosaka K., Mohammed M., Handberg E. M., Richards E. M., Pepine C. J., and Raizada M. K. (2018) Imbalance of gut microbiome and intestinal epithelial barrier dysfunction in patients with high blood pressure. Clin. Sci. 132, 701–718 10.1042/CS20180087 29507058 PMC5955695

[2] Hooper L. V., Littman D. R., and Macpherson A. J. (2012) Interactions between the microbiota and the immune system. Science 336, 1268–1273 10.1126/science.1223490 22674334 PMC4420145

[3] Trompette A., Gollwitzer E. S., Yadava K., Sichelstiel A. K., Sprenger N., Ngom-Bru C., Blanchard C., Junt T., Nicod L. P., Harris N. L., and Marsland B. J. (2014) Gut microbiota metabolism of dietary fiber influences allergic airway disease and hematopoiesis. Nat. Med. 20, 159–166 10.1038/nm.3444 24390308

[4] O'Keefe S. J., Ou J., Aufreiter S., O'Connor D., Sharma S., Sepulveda J., Fukuwatari T., Shibata K., and Mawhinney T. (2009) Products of the colonic microbiota mediate the effects of diet on colon cancer risk. J. Nutr. 139, 2044–2048 10.3945/jn.109.104380 19741203 PMC6459055

[5] Koropatkin N. M., Cameron E. A., and Martens E. C. (2012) How glycan metabolism shapes the human gut microbiota. Nat. Rev. Microbiol. 10, 323–335 10.1038/nrmicro2746 22491358 PMC4005082

[6] El Kaoutari A., Armougom F., Gordon J. I., Raoult D., and Henrissat B. (2013) The abundance and variety of carbohydrate-active enzymes in the human gut microbiota. Nat. Rev. Microbiol. 11, 497–504 10.1038/nrmicro3050 23748339

[7] McNulty N. P., Wu M., Erickson A. R., Pan C., Erickson B. K., Martens E. C., Pudlo N. A., Muegge B. D., Henrissat B., Hettich R. L., and Gordon J. I. (2013) Effects of diet on resource utilization by a model human gut microbiota containing *Bacteroides cellulosilyticus* WH2, a symbiont with an extensive glycobiome. PLoS Biol. 11, e1001637 10.1371/journal.pbio.1001637 23976882 PMC3747994

[8] Nyman M., Asp N. G., Cummings J., and Wiggins H. (1986) Fermentation of dietary fiber in the intestinal tract–comparison between man and rat. Br. J. Nutr. 55, 487–496 10.1079/BJN19860056 2823868

[9] Porter N. T., and Martens E. C. (2017) The critical roles of polysaccharides in gut microbial ecology and physiology. Annu. Rev. Microbiol. 71, 349–369 10.1146/annurev-micro-102215-095316 28657886

[10] Vandeputte D., Falony G., Vieira-Silva S., Wang J., Sailer M., Theis S., Verbeke K., and Raes J. (2017) Prebiotic inulin-type fructans induce specific changes in the human gut microbiota. Gut 66, 1968–1974 10.1136/gutjnl-2016-313271 28213610 PMC5739857

[11] Bågenholm V., Reddy S. K., Bouraoui H., Morrill J., Kulcinskaja E., Bahr C. M., Aurelius O., Rogers T., Xiao Y., Logan D. T., Martens E. C., Koropatkin N. M., and Stålbrand H. (2017) Galactomannan catabolism conferred by a polysaccharide utilization locus of *Bacteroides ovatus*: enzyme synergy and crystal structure of a β-mannanase. J. Biol. Chem. 292, 229–243 10.1074/jbc.M116.746438 27872187 PMC5217682

[12] Ndeh D., and Gilbert H. J. (2018) Biochemistry of complex glycan depolymerisation by the human gut microbiota. FEMS Microbiol. Rev. 42, 146–164 10.1093/femsre/fuy002 29325042

[13] Martens E. C., Koropatkin N. M., Smith T. J., and Gordon J. I. (2009) Complex glycan catabolism by the human gut microbiota: the bacteroidetes Sus-like paradigm. J. Biol. Chem. 284, 24673–24677 10.1074/jbc.R109.022848 19553672 PMC2757170

[14] Grondin J. M., Tamura K., Déjean G., Abbott D. W., and Brumer H. (2017) Polysaccharide utilization loci: fueling microbial communities. J. Bacteriol. 199, e00860 10.1128/JB.00860-16 28138099 PMC5512228

[15] Cuskin F., Lowe E. C., Temple M. J., Zhu Y., Cameron E., Pudlo N. A., Porter N. T., Urs K., Thompson A. J., Cartmell A., Rogowski A., Hamilton B. S., Chen R., Tolbert T. J., Piens K., et al (2015) Human gut Bacteroidetes can utilize yeast mannan through a selfish mechanism. Nature 517, 165–169 10.1038/nature13995 25567280 PMC4978465

[16] Martens E. C., Lowe E. C., Chiang H., Pudlo N. A., Wu M., McNulty N. P., Abbott D. W., Henrissat B., Gilbert H. J., Bolam D. N., and Gordon J. I. (2011) Recognition and degradation of plant cell wall polysaccharides by two human gut symbionts. PLoS Biol. 9, e1001221 10.1371/journal.pbio.1001221 22205877 PMC3243724

[17] Bjursell M. K., Martens E. C., and Gordon J. I. (2006) Functional genomic and metabolic studies of the adaptations of a prominent adult human gut symbiont, *Bacteroides thetaiotaomicron*, to the suckling period. J. Biol. Chem. 281, 36269–36279 10.1074/jbc.M606509200 16968696

[18] Dea I. C., and Morrison A. (1975) in Advances in Carbohydrate Chemistry and Biochemistry (Tipson R. S., and Horton D., eds) Vol. 31, pp. 241–312, Academic Press, New York

[19] Barak S., and Mudgil D. (2014) Locust bean gum: processing, properties and food applications–a review. Int. J. Biol. Macromol. 66, 74–80 10.1016/j.ijbiomac.2014.02.017 24548746

[20] Mudgil D., Barak S., and Khatkar B. S. (2014) Guar gum: processing, properties and food applications–a review. J. Food. Sci. Technol. 51, 409–418 10.1007/s13197-011-0522-x 24587515 PMC3931889

[21] Meier H. (1958) On the structure of cell walls and cell wall mannans from ivory nuts and from dates. Biochim. Biophys. Acta 28, 229–240 10.1016/0006-3002(58)90468-2 13535718

[22] Albrecht S., van Muiswinkel G. C., Xu J., Schols H. A., Voragen A. G., and Gruppen H. (2011) Enzymatic production and characterization of konjac glucomannan oligosaccharides. J. Agric. Food Chem. 59, 12658–12666 10.1021/jf203091h 22017574

[23] Lundqvist J., Teleman A., Junel L., Dahlman O., Zacchi G., Tjerneld F., and Stalbrand H. (2002) Isolation and characterization of galactoglucomannan from spruce (*Picea abies*). Carbohyd. Polym. 48, 29–39 10.1016/S0144-8617(01)00210-7

[24] Gilbert H. J., Stålbrand H., and Brumer H. (2008) How the walls come crumbling down: recent structural biochemistry of plant polysaccharide degradation. Curr. Opin. Plant Biol. 11, 338–348 10.1016/j.pbi.2008.03.004 18430603

[25] Lombard V., Golaconda Ramulu H., Drula E., Coutinho P. M., and Henrissat B. (2014) The carbohydrate-active enzymes database (CAZy) in 2013. Nucleic Acids Res. 42, D490–D495 10.1093/nar/gkt1178 24270786 PMC3965031

[26] Davies G. J., Wilson K. S., and Henrissat B. (1997) Nomenclature for sugar-binding subsites in glycosyl hydrolases. Biochem. J. 321, 557–559 10.1042/bj3210557 9020895 PMC1218105

[27] Malgas S., van Dyk S. J., and Pletschke B. I. (2015) β-Mannanase (Man26A) and α-galactosidase (Aga27A) synergism–a key factor for the hydrolysis of galactomannan substrates. Enzyme Microb. Technol. 70, 1–8 10.1016/j.enzmictec.2014.12.007 25659626

[28] von Freiesleben P., Spodsberg N., Blicher T. H., Anderson L., Jørgensen H., Stålbrand H., Meyer A. S., and Krogh K. B. (2016) An *Aspergillus nidulans* GH26 endo-β-mannanase with a novel degradation pattern on highly substituted galactomannans. Enzyme Microb. Technol. 83, 68–77 10.1016/j.enzmictec.2015.10.011 26777252

[29] Hekmat O., Lo Leggio L., Rosengren A., Kamarauskaite J., Kolenova K., and Stålbrand H. (2010) Rational engineering of mannosyl binding in the distal glycone subsites of *Cellulomonas fimi* endo-β-1,4-mannanase: mannosyl binding promoted at subsite −2 and demoted at subsite −3. Biochemistry 49, 4884–4896 10.1021/bi100097f 20426480

[30] Morrill J., Kulcinskaja E., Sulewska A. M., Lahtinen S., Stålbrand H., Svensson B., and Abou Hachem M. (2015) The GH5 1,4-β-mannanase from *Bifidobacterium animalis* subsp. *lactis* Bl-04 possesses a low-affinity mannan-binding module and highlights the diversity of mannanolytic enzymes. BMC Biochem. 16, 26 10.1186/s12858-015-0055-4 26558435 PMC4642672

[31] Hsu Y., Koizumi H., Otagiri M., Moriya S., and Arioka M. (2018) Trp residue at subsite −5 plays a critical role in the substrate binding of two protistan GH26 β-mannanases from a termite hindgut. Appl. Microbiol. Biotechnol. 102, 1737–1747 10.1007/s00253-017-8726-2 29305697

[32] Cartmell A., Topakas E., Ducros V. M., Suits M. D., Davies G. J., and Gilbert H. J. (2008) The *Cellvibrio japonicus* mannanase CjMan26C displays a unique exo-mode of action that is conferred by subtle changes to the distal region of the active site. J. Biol. Chem. 283, 34403–34413 10.1074/jbc.M804053200 18799462 PMC2662245

[33] Tsukagoshi H., Nakamura A., Ishida T., Touhara K. K., Otagiri M., Moriya S., Samejima M., Igarashi K., Fushinobu S., Kitamoto K., and Arioka M. (2014) Structural and biochemical analyses of glycoside hydrolase family 26 β-mannanase from a symbiotic protist of the termite *Reticulitermes speratus*. J. Biol. Chem. 289, 10843–10852 10.1074/jbc.M114.555383 24570006 PMC4036197

[34] Tailford L. E., Ducros V. M., Flint J. E., Roberts S. M., Morland C., Zechel D. L., Smith N., Bjørnvad M. E., Borchert T. V., Wilson K. S., Davies G. J., and Gilbert H. J. (2009) Understanding how diverse β-mannanases recognize heterogeneous substrates. Biochemistry 48, 7009–7018 10.1021/bi900515d 19441796

[35] Yan X. X., An X. M., Gui L. L., and Liang D. C. (2008) From structure to function: insights into the catalytic substrate specificity and thermostability displayed by *Bacillus subtilis* mannanase BCman. J. Mol. Biol. 379, 535–544 10.1016/j.jmb.2008.03.068 18455734

[36] Le Nours J., Anderson L., Stoll D., Stålbrand H., and Lo Leggio L. (2005) The structure and characterization of a modular endo-β-1,4-mannanase from *Cellulomonas fimi*. Biochemistry 44, 12700–12708 10.1021/bi050779v 16171384

[37] Ducros V. M., Zechel D. L., Murshudov G. N., Gilbert H. J., Szabó L., Stoll D., Withers S. G., and Davies G. J. (2002) Substrate distortion by a β-mannanase: snapshots of the Michaelis and covalent-intermediate complexes suggest a B(2,5) conformation for the transition state. Angew. Chem. Int. Ed. Engl. 41, 2824–2827 10.1002/1521-3773(20020802)41:15<2824::AID-ANIE2824>3.0.CO;2-G 12203498

[38] Taylor E. J., Goyal A., Guerreiro C. I., Prates J. A., Money V. A., Ferry N., Morland C., Planas A., Macdonald J. A., Stick R. V., Gilbert H. J., Fontes C. M., and Davies G. J. (2005) How family 26 glycoside hydrolases orchestrate catalysis on different polysaccharides- Structure and activity of a *Clostridium thermocellum* lichenase, CtLic26A. J. Biol. Chem. 280, 32761–32767 10.1074/jbc.M506580200 15987675

[39] Couturier M., Roussel A., Rosengren A., Leone P., Stålbrand H., and Berrin J. G. (2013) Structural and biochemical analyses of glycoside hydrolase families 5 and 26 β-(1,4)-mannanases from *Podospora anserina* reveal differences upon manno-oligosaccharide catalysis. J. Biol. Chem. 288, 14624–14635 10.1074/jbc.M113.459438 23558681 PMC3656314

[40] Kulcinskaja E., Rosengren A., Ibrahim R., Kolenová K., and Stålbrand H. (2013) Expression and characterization of a *Bifidobacterium adolescentis* β-mannanase carrying mannan-binding and cell association motifs. Appl. Environ. Microbiol. 79, 133–140 10.1128/AEM.02118-12 23064345 PMC3536085

[41] Kawaguchi K., Senoura T., Ito S., Taira T., Ito H., Wasaki J., and Ito S. (2014) The mannobiose-forming exo-mannanase involved in a new mannan catabolic pathway in *Bacteroides fragilis*. Arch. Microbiol. 196, 17–23 10.1007/s00203-013-0938-y 24217874

[42] Zhang M., Chekan J. R., Dodd D., Hong P. Y., Radlinski L., Revindran V., Nair S. K., Mackie R. I., and Cann I. (2014) Xylan utilization in human gut commensal bacteria is orchestrated by unique modular organization of polysaccharide-degrading enzymes. Proc. Natl. Acad. Sci. U.S.A. 111, E3708–E3717 10.1073/pnas.1406156111 25136124 PMC4156774

[43] Reddy S. K., Bågenholm V., Pudlo N. A., Bouraoui H., Koropatkin N. M., Martens E. C., and Stålbrand H. (2016) A β-mannan utilization locus in *Bacteroides ovatus* involves a GH36 α-galactosidase active on galactomannans. FEBS Lett. 590, 2106–2118 10.1002/1873-3468.12250 27288925 PMC5094572

[44] Hogg D., Woo E. J., Bolam D. N., McKie V. A., Gilbert H. J., and Pickersgill R. W. (2001) Crystal structure of mannanase 26A from *Pseudomonas cellulosa* and analysis of residues involved in substrate binding. J. Biol. Chem. 276, 31186–31192 10.1074/jbc.M010290200 11382747

[45] Couturier M., Haon M., Coutinho P. M., Henrissat B., Lesage-Meessen L., and Berrin J. G. (2011) Podospora anserina hemicellulases potentiate the *Trichoderma reesei* secretome for saccharification of lignocellulosic biomass. Appl. Environ. Microbiol. 77, 237–246 10.1128/AEM.01761-10 21037302 PMC3019743

[46] Mccleary B. V., and Matheson N. K. (1974) α-d-galactosidase activity and galactomannan and galactosylsucrose oligosaccharide depletion in germinating legume seeds. Phytochemistry 13, 1747–1757 10.1016/0031-9422(74)85084-3

[47] Wu H., Montanier C. Y., and Dumon C. (2017) in Protein–Carbohydrate Interactions: Methods and Protocols (Abbott D. W., and Lammerts van Bueren A., eds) pp. 129–141, Springer, New York

[48] Movahedin M., Brooks T. M., Supekar N. T., Gokanapudi N., Boons G. J., and Brooks C. L. (2017) Glycosylation of MUC1 influences the binding of a therapeutic antibody by altering the conformational equilibrium of the antigen. Glycobiology 27, 677–687 10.1093/glycob/cww131 28025250 PMC5881634

[49] Schrödinger, LLC (2015) The PyMOL Molecular Graphics System, Version 1.7, Schrödinger, LLC, New York

[50] Bolam D. N., Hughes N., Virden R., Lakey J. H., Hazlewood G. P., Henrissat B., Braithwaite K. L., and Gilbert H. J. (1996) Mannanase A from *Pseudomonas fluorescens* ssp. *cellulosa* is a retaining glycosyl hydrolase in which E212 and E320 are the putative catalytic residues. Biochemistry 35, 16195–16204 10.1021/bi961866d 8973192

[51] Kumagai Y., Usuki H., Yamamoto Y., Yamasato A., Arima J., Mukaihara T., and Hatanaka T. (2011) Characterization of calcium ion sensitive region for β-mannanase from *Streptomyces thermolilacinus*. Biochim. Biophys. Acta 1814, 1127–1133 10.1016/j.bbapap.2011.04.017 21601016

[52] Kumagai Y., Kawakami K., Mukaihara T., Kimura M., and Hatanaka T. (2012) The structural analysis and the role of calcium-binding site for thermal stability in mannanase. Biochimie 94, 2783–2790 10.1016/j.biochi.2012.09.012 23009928

[53] Srivastava P. K., Appu Rao G., A. R., and Kapoor M. (2016) Metal-dependent thermal stability of recombinant endo-mannanase (ManB-1601) belonging to family GH 26 from *Bacillus* sp. CFR1601. Enzyme Microb. Technol. 84, 41–49 10.1016/j.enzmictec.2015.12.010 26827773

[54] Wernersson S., Bagenholm V., Persson C., Upadhyay S. K., Stalbrand H., and Akke M. (2019) Backbone ^1^H, ^13^C, and ^15^N resonance assignments of BoMan26A, a β-mannanase of the glycoside hydrolase family 26 from the human gut bacterium *Bacteroides ovatus*. Biomol. NMR Assign. 13, 213–218 10.1007/s12104-019-09879-w 30734154 PMC6439179

[55] Wang K., Pereira G. V., Cavalcante J. J., Zhang M., Mackie R., and Cann I. (2016) *Bacteroides intestinalis* DSM 17393, a member of the human colonic microbiome, upregulates multiple endoxylanases during growth on xylan. Sci. Rep. 6, 34360 10.1038/srep34360 27681607 PMC5041131

[56] Armstrong Z., Mewis K., Liu F., Morgan-Lang C., Scofield M., Durno E., Chen H. M., Mehr K., Withers S. G., and Hallam S. J. (2018) Metagenomics reveals functional synergy and novel polysaccharide utilization loci in the *Castor canadensis* fecal microbiome. ISME J. 12, 2757–2769 10.1038/s41396-018-0215-9 30013164 PMC6193987

[57] Phansopa C., Roy S., Rafferty J. B., Douglas C. W., Pandhal J., Wright P. C., Kelly D. J., and Stafford G. P. (2014) Structural and functional characterization of NanU, a novel high-affinity sialic acid-inducible binding protein of oral and gut-dwelling Bacteroidetes species. Biochem. J. 458, 499–511 10.1042/BJ20131415 24351045 PMC3969230

[58] Koropatkin N. M., Martens E. C., Gordon J. I., and Smith T. J. (2008) Starch catabolism by a prominent human gut symbiont is directed by the recognition of amylose helices. Structure 16, 1105–1115 10.1016/j.str.2008.03.017 18611383 PMC2563962

[59] Glenwright A. J., Pothula K. R., Bhamidimarri S. P., Chorev D. S., Baslé A., Firbank S. J., Zheng H., Robinson C. V., Winterhalter M., Kleinekathöfer U., Bolam D. N., and van den Berg B. (2017) Structural basis for nutrient acquisition by dominant members of the human gut microbiota. Nature 541, 407–411 10.1038/nature20828 28077872 PMC5497811

[60] Cho K. H., and Salyers A. A. (2001) Biochemical analysis of interactions between outer membrane proteins that contribute to starch utilization by *Bacteroides thetaiotaomicron*. J. Bacteriol. 183, 7224–7230 10.1128/JB.183.24.7224-7230.2001 11717282 PMC95572

[61] Rogowski A., Briggs J. A., Mortimer J. C., Tryfona T., Terrapon N., Lowe E. C., Baslé A., Morland C., Day A. M., Zheng H., Rogers T. E., Thompson P., Hawkins A. R., Yadav M. P., Henrissat B., et al (2015) Glycan complexity dictates microbial resource allocation in the large intestine. Nat. Commun. 6, 7481 10.1038/ncomms8481 26112186 PMC4491172

[62] Larsbrink J., Rogers T. E., Hemsworth G. R., McKee L. S., Tauzin A. S., Spadiut O., Klinter S., Pudlo N. A., Urs K., Koropatkin N. M., Creagh A. L., Haynes C. A., Kelly A. G., Cederholm S. N., Davies G. J., et al (2014) A discrete genetic locus confers xyloglucan metabolism in select human gut Bacteroidetes. Nature 506, 498–502 10.1038/nature12907 24463512 PMC4282169

[63] von Freiesleben P., Moroz O. V., Blagova E., Wiemann M., Spodsberg N., Agger J. W., Davies G. J., Wilson K. S., Stålbrand H., Meyer A. S., and Krogh K. B. R. M. (2019) Crystal structure and substrate interactions of an unusual fungal non-CBM carrying GH26 endo-β-mannanase from *Yunnania penicillata*. Sci. Rep. 9, 2266 10.1038/s41598-019-38602-x 30783168 PMC6381184

[64] Hudson K. L., Bartlett G. J., Diehl R. C., Agirre J., Gallagher T., Kiessling L. L., and Woolfson D. N. (2015) Carbohydrate–aromatic interactions in proteins. J. Am. Chem. Soc. 137, 15152–15160 10.1021/jacs.5b08424 26561965 PMC4676033

[65] Aspeborg H., Coutinho P. M., Wang Y., Brumer H. 3rd, and Henrissat B. (2012) Evolution, substrate specificity and subfamily classification of glycoside hydrolase family 5 (GH5). BMC Evol. Biol. 12, 186 10.1186/1471-2148-12-186 22992189 PMC3526467

[66] Stålbrand H., Siikaaho M., Tenkanen M., and Viikari L. (1993) Purification and characterization of 2 β-mannanases from *Trichoderma reesei*. J. Biotechnol. 29, 229–242 10.1016/0168-1656(93)90055-R

[67] Gasteiger E., Hoogland C., Gattiker A., Duvaud S., Wilkins M. R., Appel R. D., and Bairoch A. (2005) in The Proteomics Protocols Handbook (Walker J. M., ed) pp. 571–607, Humana Press, Totowa, NJ

[68] Kabsch W. (2010) XDS. Acta Crystallogr. D Biol. Crystallogr. 66, 125–132 10.1107/S0907444909047337 20124692 PMC2815665

[69] Winn M. D., Ballard C. C., Cowtan K. D., Dodson E. J., Emsley P., Evans P. R., Keegan R. M., Krissinel E. B., Leslie A. G., McCoy A., McNicholas S. J., Murshudov G. N., Pannu N. S., Potterton E. A., Powell H. R., et al (2011) Overview of the CCP4 suite and current developments. Acta Crystallogr. D Biol. Crystallogr. 67, 235–242 10.1107/S0907444910045749 21460441 PMC3069738

[70] Keegan R. M., and Winn M. D. (2007) Automated search-model discovery and preparation for structure solution by molecular replacement. Acta Crystallogr. D Biol. Crystallogr. 63, 447–457 10.1107/S0907444907002661 17372348

[71] McCoy A. J., Grosse-Kunstleve R. W., Adams P. D., Winn M. D., Storoni L. C., and Read R. J. (2007) Phaser crystallographic software. J. Appl. Crystallogr. 40, 658–674 10.1107/S0021889807021206 19461840 PMC2483472

[72] Adams P. D., Afonine P. V., Bunkóczi G., Chen V. B., Davis I. W., Echols N., Headd J. J., Hung L. W., Kapral G. J., Grosse-Kunstleve R. W., McCoy A. J., Moriarty N. W., Oeffner R., Read R. J., Richardson D. C., et al (2010) PHENIX: a comprehensive Python-based system for macromolecular structure solution. Acta Crystallogr. D Biol. Crystallogr. 66, 213–221 10.1107/S0907444909052925 20124702 PMC2815670

[73] Emsley P., Lohkamp B., Scott W. G., and Cowtan K. (2010) Features and development of Coot. Acta Crystallogr. D Biol. Crystallogr. 66, 486–501 10.1107/S0907444910007493 20383002 PMC2852313

[74] Jones D. T., Taylor W. R., and Thornton J. M. (1992) The rapid generation of mutation data matrices from protein sequences. Comput. Appl. Biosci. 8, 275–282 1633570 10.1093/bioinformatics/8.3.275

[75] Kumar S., Stecher G., Li M., Knyaz C., and Tamura K. (2018) MEGA X: molecular evolutionary genetics analysis across computing platforms. Mol. Biol. Evol. 35, 1547–1549 10.1093/molbev/msy096 29722887 PMC5967553

[76] Wallace A. C., Laskowski R. A., and Thornton J. M. (1995) Ligplot–a program to generate schematic diagrams of protein ligand interactions. Protein Eng. 8, 127–134 10.1093/protein/8.2.127 7630882

[77] Li W., Cowley A., Uludag M., Gur T., McWilliam H., Squizzato S., Park Y. M., Buso N., and Lopez R. (2015) The EMBL-EBI bioinformatics web and programmatic tools framework. Nucleic Acids Res. 43, W580–W584 25845596 10.1093/nar/gkv279 PMC4489272

