# Supplementary material for: A surface-exposed GH26 β-mannanase from Bacteroides ovatus: Structure, role, and phylogenetic analysis of BoMan26B
Source: J Biol Chem. 2019 Apr 18;294(23):9100–17. doi: 10.1074/jbc.RA118.007171 (PMC6556568; doi:10.1074/jbc.RA118.007171)
Supplement: Supporting Information [file supp_294_23_9100__index.html]

A surface exposed GH26 β-mannanase from Bacteroides ovatus: structure, role and phylogenetic analysis of BoMan26B — A surface exposed GH26 β-mannanase from B. ovatus — A surface-exposed GH26 β-mannanase from Bacteroides ovatus: Structure, role, and phylogenetic analysis of BoMan26B — Surface-exposed GH26 β-mannanase from B. ovatus — Supporting Information 

# A surface-exposed GH26 β-mannanase from *Bacteroides ovatus*: Structure, role, and phylogenetic analysis of *Bo*Man26B

## Supporting Information

- Supporting Information to Bagenholm et al - Michaelis-Menten curves, MST control data, BoMan26B sequence and metal binding site, sequence alignment, primer sequences and SDS-PAGE
