# Supplementary material for: A surface-exposed GH26 β-mannanase from Bacteroides ovatus: Structure, role, and phylogenetic analysis of BoMan26B
Source: J Biol Chem. 2019 Apr 18;294(23):9100–17. doi: 10.1074/jbc.RA118.007171 (PMC6556568; doi:10.1074/jbc.RA118.007171)
Supplement: Supporting Information [file supp_RA118.007171_142599_2_supp_316492_pq3sw6.docx]

**Supporting information**

**
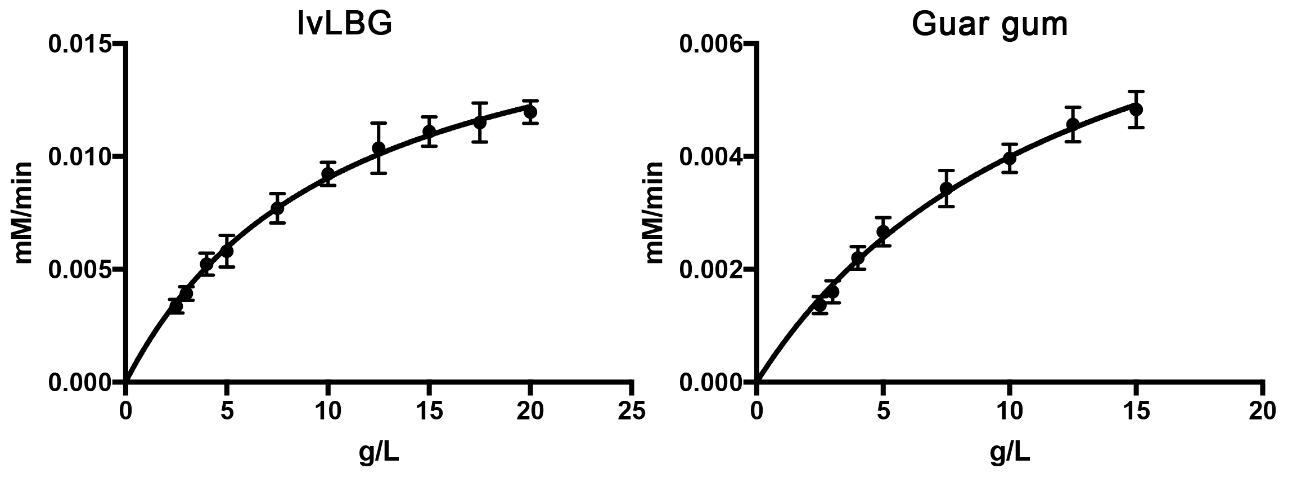
**

**Figure S1** Michaelis-Menten kinetics of *Bo*Man26B on low viscosity LBG (left) and medium viscosity guar gum (right), generated in GraphPad Prism 6 (La Jolla, California, USA)

A

B

**Figure S2** Control experiments for the MST analysis of the SusD-like protein and G2M5 (ligand). *A*: the SD-test, showing fluorescence for SusD-like protein boiled in the presence of SDS and DTT with increasing ligand concentration. *B*: The fluorescence of fluorescently labeled *Tm*NrdD, which does not bind G2M5, with increasing ligand concentration.


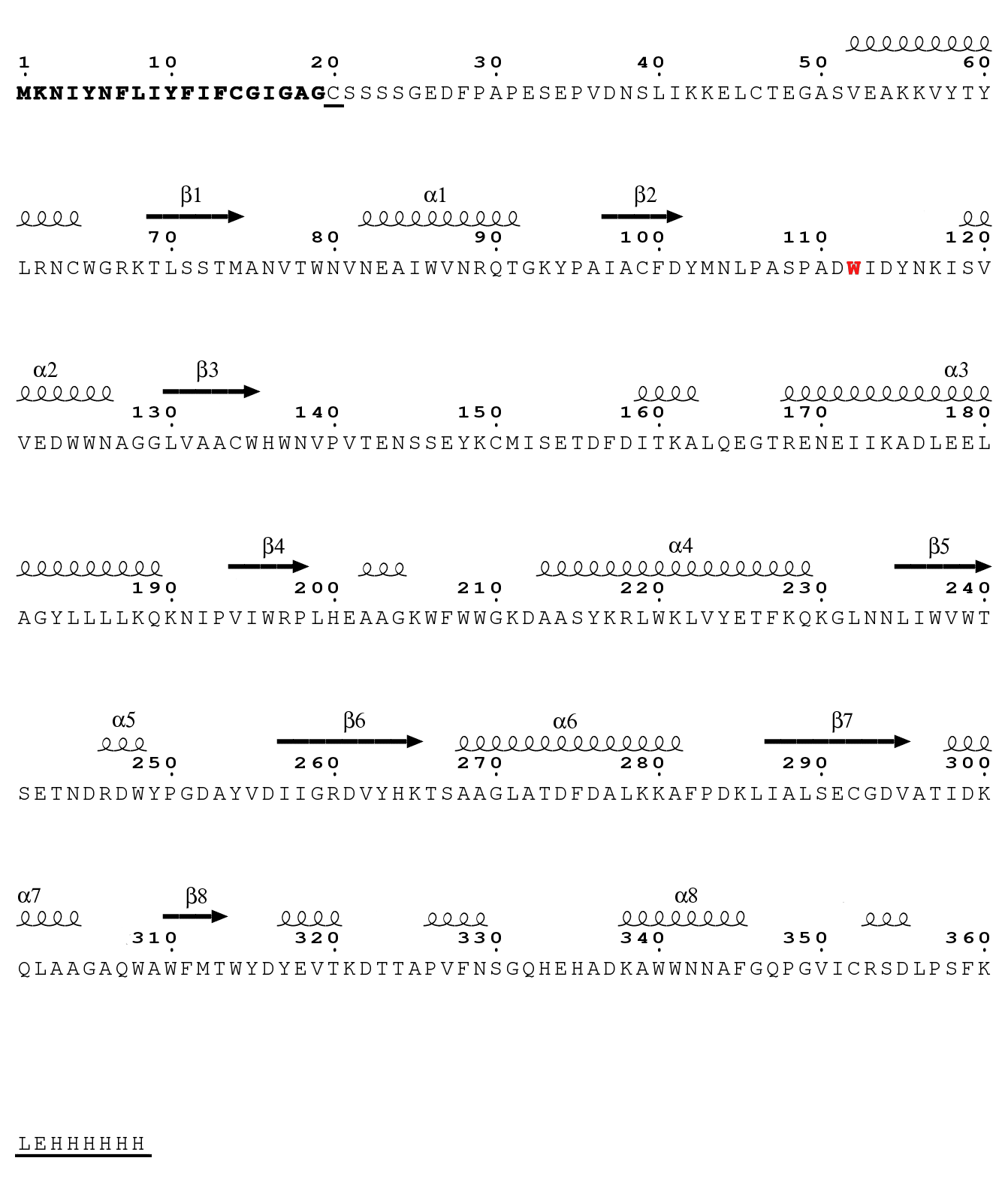


**Figure S3** The native sequence of *Bo*Man26B, also showing the attached His-tag (underlined), where the Leu codon replaced the native stop codon. An initial signal peptide sequence (bold) was not included in the expressed construct, which begins with a predicted lipid anchoring cysteine (underlined). The secondary structure elements are shown and W112 located in the –5 subsite is colored red. The catalytic residues are E201 (acid/base) and E291 (nucleophile).

**
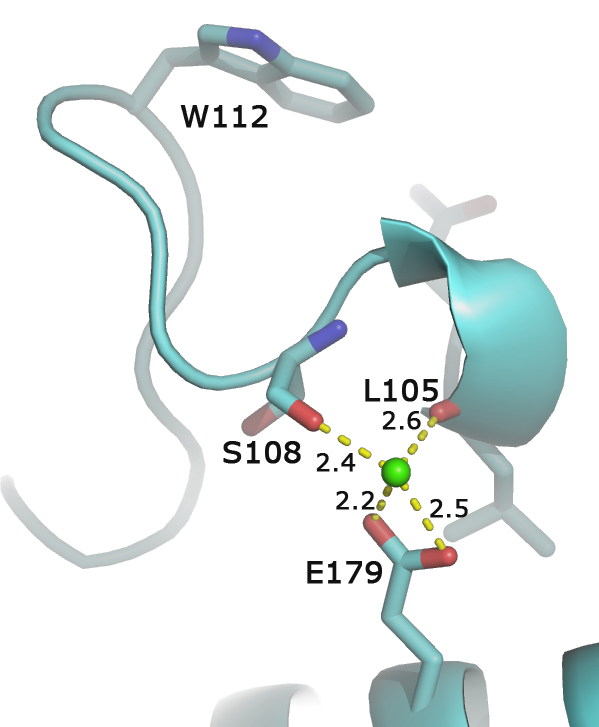
**

**Figure S4** The calcium site in *Bo*Man26B, showing the distances to the surrounding residues in Å. The loop connecting the metal site with W112 in the –5 subsite is also shown.

B._cellulosilyticus_26A ---------------------------------------------------------------- 0

B._xylanisolvens_NLAE-zl-P352_26A ---------------------------------------------------------------- 0

BoMan26A ---------------------------------------------------------------- 0

B._ovatus_CL02T12C04_26A ---------------------------------------------------------------- 0

B._salanitronis_26A ---------------------------------------------------------------- 0

B._sp._3_1_23_26A ---------------------------------------------------------------- 0

B._ovatus_CL03T12C18_26A ---------------------------------------------------------------- 0

B._sp._D2_26A ---------------------------------------------------------------- 0

RsMan26C ---------------------------------------------------------------- 0

Prevotella_bryantii_B14_AAC97596.1 MRKFILAFIFAICVFRAKAADKSLIITFNDNTTQIFALSDLPNIKMQNDKMTIVAGLTTAEYDL 64

uncultured_bacterium_ADA62505.1 ---------------------------------------------------------------- 0

uncultured_bacterium_ABB46200.1 ---------------------------------------------------------------- 0

B._cellulosilyticus_26B ---------------------------------------------------------------- 0

B._salanitronis_26B ---------------------------------------------------------------- 0

B._ovatus_CL03T12C18_26B ---------------------------------------------------------------- 0

B._sp._3_1_23_26B ---------------------------------------------------------------- 0

**BoMan26B ---------------------------------------------------------------- 0**

B._xylanisolvens_NLAE-zl-P352_26B ---------------------------------------------------------------- 0

B._sp._D22_26B ---------------------------------------------------------------- 0

B._cellulosilyticus_26A ---------------------------------------------------------------- 0

B._xylanisolvens_NLAE-zl-P352_26A ---------------------------------------------------------------- 0

BoMan26A ---------------------------------------------------------------- 0

B._ovatus_CL02T12C04_26A ---------------------------------------------------------------- 0

B._salanitronis_26A ---------------------------------------------------------------- 0

B._sp._3_1_23_26A ---------------------------------------------------------------- 0

B._ovatus_CL03T12C18_26A ---------------------------------------------------------------- 0

B._sp._D2_26A ---------------------------------------------------------------- 0

RsMan26C ---------------------------------------------------------------- 0

Prevotella_bryantii_B14_AAC97596.1 YKVRTFTFGESTGIEDVTNEKLTFEGNSLIIPGSSNQIRIFSINGMCVSLDSTQLGSFTVLNLE 128

uncultured_bacterium_ADA62505.1 ---------------------------------------------------------------- 0

uncultured_bacterium_ABB46200.1 ---------------------------------------------------------------- 0

B._cellulosilyticus_26B ---------------------------------------------------------------- 0

B._salanitronis_26B ---------------------------------------------------------------- 0

B._ovatus_CL03T12C18_26B ---------------------------------------------------------------- 0

B._sp._3_1_23_26B ---------------------------------------------------------------- 0

**BoMan26B ---------------------------------------------------------------- 0**

B._xylanisolvens_NLAE-zl-P352_26B ---------------------------------------------------------------- 0

B._sp._D22_26B ---------------------------------------------------------------- 0

B._cellulosilyticus_26A ---------------------------------------------------------------- 0

B._xylanisolvens_NLAE-zl-P352_26A ---------------------------------------------------------------- 0

BoMan26A ---------------------------------------------------------------- 0

B._ovatus_CL02T12C04_26A ---------------------------------------------------------------- 0

B._salanitronis_26A ---------------------------------------------------------------- 0

B._sp._3_1_23_26A ---------------------------------------------------------------- 0

B._ovatus_CL03T12C18_26A ---------------------------------------------------------------- 0

B._sp._D2_26A ---------------------------------------------------------------- 0

RsMan26C ---------------------------------------------------------------- 0

Prevotella_bryantii_B14_AAC97596.1 SLPQGVYVINTNGKSVKITKKKKFIFSLLATLISFSVFAQSADDTYVVNKADGTSQSYKVMEFP 192

uncultured_bacterium_ADA62505.1 ---------------------------------------------------------------- 0

uncultured_bacterium_ABB46200.1 ---------------------------------------------------------------- 0

B._cellulosilyticus_26B ---------------------------------------------------------------- 0

B._salanitronis_26B ---------------------------------------------------------------- 0

B._ovatus_CL03T12C18_26B ---------------------------------------------------------------- 0

B._sp._3_1_23_26B ---------------------------------------------------------------- 0

**BoMan26B ---------------------------------------------------------------- 0**

B._xylanisolvens_NLAE-zl-P352_26B ---------------------------------------------------------------- 0

B._sp._D22_26B ---------------------------------------------------------------- 0

B._cellulosilyticus_26A ---------------------**M**I**N**VI-MRNFRL---**L**TMTL-LI**G**SLVS**C**GPVQKSQ------- 31

B._xylanisolvens_NLAE-zl-P352_26A -------------------------M-MKYIILTINA**I**FCL-LL--PTA**CS**G**S**----------- 24

BoMan26A -------------------------M-MKYIILTINA**I**FCL-LL--PTA**CS**G**S**----------- 24

B._ovatus_CL02T12C04_26A -------------------------M-MKYIILTINA**I**FCL-LL--PTA**CS**G**S**----------- 24

B._salanitronis_26A ---------------------------MKPIH-TILAGTC--LIASMTA**C**APQ---Q------- 24

B._sp._3_1_23_26A ---------------------------MKMKNKLVMAGI**F**L-LALGMG**GC**VAR---E------- 26

B._ovatus_CL03T12C18_26A -----------------------------------MAGV**F**L-LALGMG**GC**VIR---E------- 18

B._sp._D2_26A -----------------------------------MAGV**F**L-LALGMG**GC**VIR---E------- 18

RsMan26C ---------------------------------------------------------------- 0

Prevotella_bryantii_B14_AAC97596.1 NIKFNGDGTFGNYMTGFDDFGQI**N**VW**N**ISDV-KSVT---**F**N-----------------I**A**H-**S**N 234

uncultured_bacterium_ADA62505.1 ---------------------------MKRL-KRNF**L**LTLLTA**C**--------------IPFMVY 22

uncultured_bacterium_ABB46200.1 ---------------------------MKHL-KSLM**L**VV**F**--VL--------------M**A**TARA 20

B._cellulosilyticus_26B ---------------------------------------------------------------- 0

B._salanitronis_26B ---------------------------------------------------------------- 0

B._ovatus_CL03T12C18_26B ------------MKKSRPTIGIVKSRV---**F**-LTGTAFVLM-A**C**--------**SSGE**E**F**AGH-**S**G 38

B._sp._3_1_23_26B ----------------------------------------M-A**C**--------**SSGE**E**F**AGH-**S**G 14

**BoMan26B ---------------------MKNIYN---F-----LIYFI-FCGIGAGCSSSSGEDFPAPESE 34**

B._xylanisolvens_NLAE-zl-P352_26B ---------------------**MKNIYN**---**F**-----**LIYFI**-**FCGIGAGCSSSSGEDFP**V**PE**P**E** 34

B._sp._D22_26B ---------------------**MKNIYN**---**F**-----**LIYFI**-**FCGIGAGCSSSSGEDFP**V**PE**P**E** 34

B._cellulosilyticus_26A --------K**E**ERSSVRTS**E**TENLLAN**L**KKVSS**R**G---FMFGHHDDTNYGIG**W**EGD**E**GRSD**V**KSV 84

B._xylanisolvens_NLAE-zl-P352_26A ----------GETGGKTP**E**TVALLQN**L**KQAERKG---ILFGHHDDTAYGIG**W**EGDKGRSD**V**KSV 75

BoMan26A ----------GETGEKTP**E**TVALLQN**L**KQAERKG---ILFGHHDDTAYGIG**W**EGDKGRSD**V**KSV 75

B._ovatus_CL02T12C04_26A ----------GETGEKTP**E**TVALLQN**L**KQAERKG---ILFGHHDDTAYGIG**W**EGDKGRSD**V**KSV 75

B._salanitronis_26A --------K**E**TPKPERTP**EA**LAMLQT**L**KTLPQQGV--FMFGHHDDPVYGIR**W**DGD**E**NRSD**V**KSV 78

B._sp._3_1_23_26A --------QQ--SASKT**VE**TEALLDK**L**IRLPEKG---FMFGHQDDPVYGIR**W**DGD**E**NRSD**V**KSV 77

B._ovatus_CL03T12C18_26A --------QQ--SASKTA**E**TEALLDK**L**IRLPKKG---FMFGHQDDPVYGIR**W**DGD**E**NRSD**V**KSV 69

B._sp._D2_26A --------QQ--SASKTA**E**TEALLDK**L**IRLPKKG---FMFGHQDDPVYGIR**W**DGD**E**NRSD**V**KSV 69

RsMan26C --QDWN**I**SSSPV**T**PSPSAG**A**Q**K**L**Y**SF**L**VQNFQK**K**II**S**GA**M**TLQGGD---ESAQTK**E**-PD**W**LQQN 58

Prevotella_bryantii_B14_AAC97596.1 **PVD**V**S**G--VF**L**ADAS**A**NDA**AKK**L**Y**K**YLR**LVY**G**N**K**I**LS**GM**MA**H-------**V**A**WN**HD**E**-**A**DKIHVL 288

uncultured_bacterium_ADA62505.1 AA**D**IP----STPVTASTDA**AK**NL**Y**A**Y**FLDQY**G**K**KT**I**SS**V**MAN**-------**V**N**WN**NTC-**A**EK**V**YKL 74

uncultured_bacterium_ABB46200.1 RA**D**I**S**----TTPVTASTDA**AK**NL**Y**A**Y**FLDQY**G**K**KT**I**SS**V**MAN**-------**V**N**WN**NTC-**A**EK**V**YKL 72

B._cellulosilyticus_26B ----VALDKS**L**VNAS**A**TPA**A**Q**KVY**A**YL**LENFES**KTLS**AM**MAN**-------**V**N**WN**TEK-SEQ**V**YQW 52

B._salanitronis_26B ---TTGLNTTPV-NSLTA**EA**QQ**VY**DF**L**VENFETR**TLS**A**TMA**VDGVSGQTGS**WN**TAD-**A**EQ**V**YQW 59

B._ovatus_CL03T12C18_26B KGEEPV**I**TPV**LCT**S**GAS**EQ**A**V**KVY**DF**LR**ENRNK**KTLS**G**TMA**C-------PS**WNVNE**-**A**E**WV**YQH 94

B._sp._3_1_23_26B KGEEPV**I**TPV**LCT**S**GAS**EQ**A**V**KVY**DF**LR**ENRNK**KTLS**G**TMA**C-------PS**WNVNE**-**A**E**WV**YQH 70

**BoMan26B PVDNSLIKKELCTEGASVEAKKVYTYLRNCWGRKTLSSTMAN-------VTWNVNE-AIWVNRQ 90**

B._xylanisolvens_NLAE-zl-P352_26B **PVDNSLIKKELCTEGASVEAKKVYTYLRNCWGRKTLSSTMAN**-------**V**A**WNVNE**-**AIWVNRQ** 90

B._sp._D22_26B **PVDNSLIKKELCTEGASVEAKKVYTYLRNCWGRKTLSSTMAN**-------**V**A**WNVNE**-**AIWVNRQ** 90

. : : : : : :

B._cellulosilyticus_26A C**G**D**YPA**VIS**FD**LGHIELG---DTMSL**D**---KVPFT**K**IRKEILNQYKR**GG**MSSLS**WH**LRN**P**L**T**GG 142

B._xylanisolvens_NLAE-zl-P352_26A C**G**A**YP**GVMS**FD**LGEIELG---GTHNL**D**---KVSFAHLREYIIEQYAR**GG**MISLS**WH**VRN**P**K**T**GG 133

BoMan26A C**G**A**YP**GVMS**FD**LGEIELG---GTHNL**D**---KVSFAHLREYIIEQYAR**GG**MISLS**WH**VRN**P**K**T**GG 133

B._ovatus_CL02T12C04_26A C**G**A**YP**GVMS**FD**LGEIELG---GTHNL**D**---KVSFAHLREYIIEQYAR**GG**MISLS**WH**VRN**P**K**T**GG 133

B._salanitronis_26A C**G**D**YPA**MMS**FD**LGHIELG---DSVNL**D**---KVSFDRIRRE**V**VAQYER**GG**MSSFS**WH**LDN**P**Q**T**GK 136

B._sp._3_1_23_26A C**G**D**YPA**VMA**FD**LGRIERG---GEKNL**D**---DVLFERIRTEIIAHYDR**GG**VCSLS**WH**VDN**PVT**GE 135

B._ovatus_CL03T12C18_26A C**G**D**YPA**VMA**FD**LGRIERG---GEKNL**D**---DVLFERIRAEIIAHY**N**R**GG**VCSLS**WH**VDN**PVT**GE 127

B._sp._D2_26A C**G**D**YPA**VMA**FD**LGRIERG---GEKNL**D**---DVLFERIRAEIIAHY**N**R**GG**VCSLS**WH**VDN**PVT**GE 127

RsMan26C A**G**HR**PA**LVGL**D**F**M**FQTGK---GEE**W**YYNDSRFSKQV-VNGAKSY**W**QK**GG**IP**A**L**CWHW**RD**P**SKDT 118

Prevotella_bryantii_B14_AAC97596.1 **TGKYPAI**N**C**Y**D**FIHI**A**VPNQGSNG**WI**N------**Y**ND-ITP**V**TE**W**AD**AGG**I**V**SLM**WH**F**NVP**QN**EN** 345

uncultured_bacterium_ADA62505.1 **TGKYPA**MN**C**Y**D**FIHICF**S**---**PA**N**WID**------**Y**TD-ITP**V**K**DW**HD**AGG**I**V**QLM**WH**F**NVP**KSQG 128

uncultured_bacterium_ABB46200.1 **TGKYPA**MN**C**Y**D**FIHICF**S**---**PA**N**WID**------**Y**TD-ITPAKE**W**HD**AGG**I**V**QLM**WH**F**NVP**KSQG 126

**B**._cellulosilyticus_26B **TGKYPAI**N**CFDY**VH**L**Y**AS**---G**A**N**WI**N------**Y**SD-ITP**V**K**DWWNAGGLV**S**A**M**WHWNVP**TKAP 106

B._salanitronis_26B **TG**Q**YPA**MN**CFDY**LH**L**AS**S**---**P**SS**WID**------**Y**SD-ITP**V**KE**WW**DN**GG**I**V**L**A**M**WHWNVP**K**TE**G 113

B._ovatus_CL03T12C18_26B **TGKYPAIA**F**FDY**LS**L**EY**S**---**P**CS**WID**------**Y**S**K**-TKI**VEDWWN**KN**GLV**G**A**G**WHW**R**VP**CVQG 148

B._sp._3_1_23_26B **TGKYPAIA**F**FDY**IS**L**EY**S**---**P**CS**WID**------**Y**S**K**-TKI**VEDWWN**KN**GLV**G**A**G**WHW**R**VP**CVQG 124

**BoMan26B TGKYPAIACFDYMNLPAS---PADWID------YNK-ISVVEDWWNAGGLVAACWHWNVPVTEN 144**

B._xylanisolvens_NLAE-zl-P352_26B **TGK**H**PAIACFDYMNLPAS**---**PADWID**------**YNK**-T**SVVEDWWNAGGLVAACWHWNVPVTEN** 144

B._sp._D22_26B **TGK**H**PAIACFDYMNLPAS**---**PADWID**------**YNK**-T**SVVEDWWNAGGLVAACWHWNVPVTEN** 144

* *.: * .*: ** *

B._cellulosilyticus_26A D**S**WDVSD--------------------------------------------------------- 149

B._xylanisolvens_NLAE-zl-P352_26A D**S**WDVTD--------------------------------------------------------- 140

BoMan26A D**S**WDVTD--------------------------------------------------------- 140

B._ovatus_CL02T12C04_26A D**S**WDVTD--------------------------------------------------------- 140

B._salanitronis_26A DTWDVSD--------------------------------------------------------- 143

B._sp._3_1_23_26A A**S**WDVSD--------------------------------------------------------- 142

B._ovatus_CL03T12C18_26A A**S**WDVSD--------------------------------------------------------- 134

B._sp._D2_26A A**S**WDVSD--------------------------------------------------------- 134

RsMan26C DAF**Y**SPS--------------------------------------------------------- 125

Prevotella_bryantii_B14_AAC97596.1 TTIGADG--------------------------------------------------------- 352

uncultured_bacterium_ADA62505.1 **S**T-------------------------------------------------------------- 130

uncultured_bacterium_ABB46200.1 AT-------------------------------------------------------------- 128

B._cellulosilyticus_26B DVFSEGLWTGEQAMPGDWSGNVQLTDDAAKVAFAEAQVGNKVRVTVKDIAAGAQGSFKNSSWLE 170

B._salanitronis_26B DTDT------------------------------------------------------------ 117

B._ovatus_CL03T12C18_26B **S**A**E**RHYT-------PGD----------------------------------------------- 158

B._sp._3_1_23_26B **S**A**E**RHYT-------PGD----------------------------------------------- 134

**BoMan26B SSEYKCM-------IS------------------------------------------------ 153**

B._xylanisolvens_NLAE-zl-P352_26B **SSEYKCM**-------**IS**------------------------------------------------ 153

B._sp._D22_26B **SSEYKCM**-------**IS**------------------------------------------------ 153

B._cellulosilyticus_26A ---------------------------------------------------------------T 150

B._xylanisolvens_NLAE-zl-P352_26A ---------------------------------------------------------------S 141

BoMan26A ---------------------------------------------------------------S 141

B._ovatus_CL02T12C04_26A ---------------------------------------------------------------S 141

B._salanitronis_26A ---------------------------------------------------------------S 144

B._sp._3_1_23_26A ---------------------------------------------------------------S 143

B._ovatus_CL03T12C18_26A ---------------------------------------------------------------S 135

B._sp._D2_26A ---------------------------------------------------------------S 135

RsMan26C --------------------------------------------------------SGNSA**T**Q**F** 133

Prevotella_bryantii_B14_AAC97596.1 ----------------------------------------------------SGQGINSSQ**T**T**F** 364

uncultured_bacterium_ADA62505.1 -----------------------------------------------------DFTCTPS**ET**T**F** 141

uncultured_bacterium_ABB46200.1 -----------------------------------------------------DVTCTPS**ET**T**F** 139

B._cellulosilyticus_26B IAPGMDYFDITGDFELEITEAVLTSLKDGGLIIGGHDYTVTGVYLEGGSASDTKYAFYRED**TDF** 234

B._salanitronis_26B ----------------------------------------------------NNYTSTLS**ET**A**F** 129

B._ovatus_CL03T12C18_26B -------------------------------------------------GSVDPGTGKRTT**T**V**F** 173

B._sp._3_1_23_26B -------------------------------------------------GSVDPGTGKRTT**T**V**F** 149

**BoMan26B ------------------------------------------------------------ETDF 157**

B._xylanisolvens_NLAE-zl-P352_26B ------------------------------------------------------------**ETDF** 157

B._sp._D22_26B ------------------------------------------------------------**ETDF** 157

B._cellulosilyticus_26A TVVKSILP**G**GANH**E**KFTGWVSKVSAFINS**L**QTEEGVKV**PV**LF**RP**W**HE**HT**G**------------S**W** 202

B._xylanisolvens_NLAE-zl-P352_26A TVVASVMQ**G**GENHVKMLEWIDRV**A**DF**LL**S**LK**TKEGVL**IPV**VF**RP**W**HE**HT**G**------------S**W** 193

BoMan26A TVVASVMQ**G**GENHVKMLEWIDRV**A**DF**LL**S**LK**TKEGVL**IPV**VF**RP**W**HE**HT**G**------------S**W** 193

B._ovatus_CL02T12C04_26A TVVASVMQ**G**GENHVKMLEWIDRV**A**DF**LL**S**LK**TKEGVL**IPV**VF**RP**W**HE**HT**G**------------S**W** 193

B._salanitronis_26A TVVRSVLP**G**GANH**E**KFITW**L**DRA**A**D**Y**MNSIQTADGTKV**PV**LF**RP**W**HE**HT**G**------------S**W** 196

B._sp._3_1_23_26A TAVASVLP**G**GENHGKFLGW**L**DRV**A**DFMNS**L**VTKEGVK**IPVI**F**RP**W**HE**HT**G**------------S**W** 195

B._ovatus_CL03T12C18_26A TAVASVLP**G**GENHGKFLGW**L**DRV**A**DFMNS**L**VTKEGVK**IPVI**F**RP**W**HE**HT**G**------------S**W** 187

B._sp._D2_26A TAVASVLP**G**GENHGKFLGW**L**DRV**A**DFMNS**L**VTKEGVK**IPVI**F**RP**W**HE**HT**G**------------S**W** 187

RsMan26C **D**ADQ**A**VKS**GT**A**EN**KA**I**LQ**DL**AVI**A**DQ**L**QD**L**R---DAGVA**V**L**WRPLHEA**S**G**------------**KW** 182

Prevotella_bryantii_B14_AAC97596.1 KASH**AL**VS**GT**W**EN**KFFMEQMENV**A**NVI**L**K**L**Q---DAG**I**VAL**WRP**F**HEAAG**NATLKSGANWGKA**W** 425

uncultured_bacterium_ADA62505.1 KASN**AL**VS**GT**W**EN**TWFYEQMDKV**A**ASI**L**K**L**Q---EAG**I**AAT**WRP**F**HEAAG**NACAKQQADWTKA**W** 202

uncultured_bacterium_ABB46200.1 KASN**AL**VS**GT**W**EN**KWFYEQMDKVIATI**L**K**L**Q---DAG**I**AAT**WRP**F**HEAAG**NACAKQQADWTKA**W** 200

B._cellulosilyticus_26B DADN**AL**T**EGT**W**EN**KVFTE**DL**AKV**A**AN**L**K**LL**Q---DEG**IPVIWRP**F**HEAAG**------------G**W** 283

B._salanitronis_26B NIDN**A**FT**EGT**W**E**YRTVQ**ADLE**KM**AGYL**K**LL**H---DA**NIPVIWRPLHEAAG**------------G**W** 178

B._ovatus_CL03T12C18_26B SAAN**A**TK**EGT**W**ENE**VV**KADL**KK**LAGYL**K**LL**R---D**K**R**IPVIWRPLHEAAG**NIYNY---KNGKA**W** 231

B._sp._3_1_23_26B SAAN**A**TK**EGT**W**ENE**VV**KADL**KK**LAGYL**K**LL**R---D**K**R**IPVIWRPLHEAAG**NIYNY---KNGKA**W** 207

**BoMan26B DITKALQEGTRENEIIKADLEELAGYLLLLK---QKNIPVIWRPLHEAAG------------KW 206**

B._xylanisolvens_NLAE-zl-P352_26B **DI**A**KALQEGTRENEIIKADLEELAGYLLLLK**---**QKNIPVIWRPLHEAAG**------------**KW** 206

B._sp._D22_26B **DI**A**KALQEGTRENEIIKADLEELAGYLLLLK**---**QKNIPVIWRPLHEAAG**------------**KW** 206

: * : . : : : : . :** ** :* *

B._cellulosilyticus_26A **FWWG**EKLCTPEE**YK**A**LW**HMTVDILR-ND**G**VD**N**ALYAYSPGSEPQ---DTAQYLKR**YPGD**ELI**D**L 262

B._xylanisolvens_NLAE-zl-P352_26A **FWWGKD**LCSSEQ**YK**T**LW**RMTNDRLR-L**KG**V**NN**VLLAYSPGMESD---TVEEYLER**YPGD**DII**D**V 253

BoMan26A **FWWGKD**LCSSEQ**YK**T**LW**RMTNDRLR-L**KG**V**NN**VLLAYSPGMESD---TVEEYLER**YPGD**DII**D**V 253

B._ovatus_CL02T12C04_26A **FWWGKD**LCSSEQ**YK**T**LW**RMTNDRLR-L**KG**V**NN**VLLAYSPGMESD---TVEEYLER**YPGD**DII**D**V 253

B._salanitronis_26A **FWWG**QKLCSTEE**YK**A**LW**SMT**Y**NRM**K**-E**KG**ADQ**L**L**W**AYSPGIEPN---DTTEYLER**YPGD**SI**VD**L 256

B._sp._3_1_23_26A **FWWG**QNLCSAEE**YK**L**LW**RMT**Y**DYLQ-E**KG**V**N**H**L**LYAYSPGSEPD---NVNEYLER**YPGD**GM**VD**L 255

B._ovatus_CL03T12C18_26A **FWWG**QNLCSAEE**YK**S**LWK**MT**Y**DYLQ-E**KG**V**N**H**L**LYAYSPGSEPD---NVNEYLER**YPGD**GM**VD**L 247

B._sp._D2_26A **FWWG**QNLCSAEE**YK**S**LWK**MT**Y**DYLQ-E**KG**V**N**H**L**LYAYSPGSEPD---NVNEYLER**YPGD**GM**VD**L 247

RsMan26C **FWWG**YKG--ADAL**K**K**LWK**IEFDY**F**VKERN**LNNLIWV**F**T**AG**T**------PIEGIA**DWYPGD**DM**VD**V 238

Prevotella_bryantii_B14_AAC97596.1 **FWWG**E**D**G--PDV**YK**Q**LW**HTMFNY**F**S-N**KG**IH**NLIW**E**WTS**QNYNGDSDIYN**ND**D**DWYPGDAYVDI** 486

uncultured_bacterium_ADA62505.1 **FWWG**Y**D**G--ADT**YK**K**LW**IAM**Y**DY**FK**-**QKG**I**NNLIW**M**WT**TQNYNGDSSKYNQ**D**TN**WYPGD**Q**YVDI** 263

uncultured_bacterium_ABB46200.1 **FWWG**Y**D**G--ADT**YK**K**LW**IAM**Y**DY**FK**-L**KG**V**NNLIW**M**WT**TQNYNGDSSKYNQ**D**T**DWYPGD**E**YVDI** 261

B._cellulosilyticus_26B **FWWGK**N**A**---T**S**F**K**NM**W**IAMFNY**FK**-AE**GLNNLIWVWT**T**ET**---------G**D**D**DWYPGDAYVDI** 334

B._salanitronis_26B **FWWGK**N**A**---D**S**F**K**K**LW**IQMFDY**FK**-AQ**GLNNLIWVWTSET**---------G**D**A**DWYPGDAYVDI** 229

B._ovatus_CL03T12C18_26B **FWWG**N**D**G--AEA**YK**K**LW**IYIFNY**FK**-KE**G**I**NNLIWVWT**TQ**T**---------K**D**SEF**YPGD**E**YVD**M 283

B._sp._3_1_23_26B **FWWG**N**D**G--AEA**YK**K**LW**IYIFNY**FK**-KE**G**I**NNLIWVWT**TQ**T**---------K**D**SEF**YPGD**E**YVD**M 259

**BoMan26B FWWGKDA---ASYKRLWKLVYETFK-QKGLNNLIWVWTSET---------NDRDWYPGDAYVDI 257**

B._xylanisolvens_NLAE-zl-P352_26B **FWWGKDA**---**ASYKRLWKLVYETFK**-**QKGLNNLIWVWTSET**---------**NDRDWYPGDAYVDI** 257

B._sp._D22_26B **FWWGKDA**---**ASYKRLWKLVYETFK**-**QKGLNNLIWVWTSET**---------**NDRDWYPGDAYVDI** 257

**** . * :* : : . .: : :: . **** :*:

B._cellulosilyticus_26A **IG**F**D**T**Y**QFERDTYLANLEKSLAIVDSIGKAHN**K**V**IA**IT**E**T**G**YEGIPDPKWWTETLLPGI-GNYP 325

B._xylanisolvens_NLAE-zl-P352_26A L**G**T**DVY**QFERSQYIKQLNKMLTILTEAGKKH**DK**P**IAL**T**E**T**G**LEGIPDSLWWTGTLLPVI-EKYP 316

BoMan26A L**G**T**DVY**QFERSQYIKQLNKMLTILTEAGKKH**DK**P**IAL**T**E**T**G**LEGIPDSLWWTGTLLPVI-EKYP 316

B._ovatus_CL02T12C04_26A L**G**T**DVY**QFERSQYIKQLNKMLTILTEAGKKH**DK**P**IA**MT**E**T**G**LEGIPDSLWWTGTLLPVI-EKYP 316

B._salanitronis_26A **IG**V**D**A**Y**QFERAAYEQALDKSLRIMTQIGKSHN**K**A**IA**VT**E**T**G**YETIPDSTWWTGTLMPII-QKYP 319

B._sp._3_1_23_26A F**G**F**D**T**Y**QFEREKYVDTMEKSLTILTEVGRLHK**K**PV**A**VT**E**T**G**YEAIPDSTWWTETLFPIV-DKYP 318

B._ovatus_CL03T12C18_26A F**G**F**D**T**Y**QFEREKYVDTMEKSLTILTEVGRLHK**K**PV**A**VT**E**T**G**YEAIPDSTWWTETLFPIV-DKYP 310

B._sp._D2_26A F**G**F**D**T**Y**QFEREKYVDTMEKSLTILTEAGRLHN**K**PV**A**VT**E**T**G**YEAIPDSTWWTETLFPIV-DKYP 310

RsMan26C **IG**M**D**I**Y**ATQGDHATQQD-YFNQCKSI--FKGR**K**IV**A**MS**ECG**S**V**PEPD-------------LAAP 286

Prevotella_bryantii_B14_AAC97596.1 **IGRD**L**Y**GT**T**AV-----Q-QYSEYSQ**LK**GRY**P**S**K**M**IAL**A**ECG**VNNSTIT----**A**DVEQAWNA**G**AK 540

uncultured_bacterium_ADA62505.1 **I**A**RD**L**Y**GYDA**A**-----Q-NLQE**F**NEIQATY**P**N**K**MVV**L**G**ECG**YGNNGDP----GKMSDVW**A**K**G**AK 317

uncultured_bacterium_ABB46200.1 VA**RD**L**Y**GYNAD-----Q-NLQE**F**SEIQA**A**Y**P**N**K**MVV**L**G**ECG**KGDSGDP----GKMSDVW**A**K**G**AK 315

B._cellulosilyticus_26B V**GRD**I**Y**T**K**DAS-----T-C**A**S**D**YSSIVV**A**YGN**K**MV**ALSECG**---------TVGK**I**SE**Q**W**A**A**G**AR 383

B._salanitronis_26B **IGRD**L**Y**GNDA**A**-----D-C**A**SQYQT**L**IND**F**GN**K**M**I**T**LSECG**YSEYTDS--TVGLLSE**Q**WNA**G**AR 285

B._ovatus_CL03T12C18_26B V**GRD**M**Y**PAKDEYTTGEY-CFRQYGTITASC**P**G**KL**V**ALSECG**NGEQSGKVYHL**A**R**I**SA**Q**WEA**G**AK 346

B._sp._3_1_23_26B V**GRD**M**Y**PAKDEYTTGEY-CFRQYGTITASC**P**G**KL**V**ALSECG**NGEQSGKVYHL**A**R**I**SA**Q**WEA**G**AK 322

**BoMan26B IGRDVYHKTSAAG-----LATDFDALKKAFPDKLIALSECGDV---------ATIDKQLAAGAQ 307**

B._xylanisolvens_NLAE-zl-P352_26B **IGRDVYHKTSAAG**-----**LA**M**DFDALKKAFPDK**M**IALSECGDV**---------**ATIDKQLA**V**G**V**Q** 307

B._sp._D22_26B **IGRDVYHKTSAAG**-----**LA**M**DFDALKKAFPDKLIALSECGDV**---------**ATIDKQLA**V**G**V**Q** 307

.. * * * :.: * *

B._cellulosilyticus_26A I**A**YVLV**W**RNARERITHFYA-------PYPGQT------------SAE**D**FMT**F**YNNPKTLFAADI 370

B._xylanisolvens_NLAE-zl-P352_26A LSYVLV**W**RNAREKSTHYYA-------PYPGQV------------SAD**D**FVK**F**SRSPKILFVGDN 361

BoMan26A LSYVLV**W**RNAREKSTHYYA-------PYPGQV------------SAD**D**FVK**F**SRSPKILFVGDN 361

B._ovatus_CL02T12C04_26A LSYVLV**W**RNAREKSTHYYA-------PYPGQV------------SAD**D**FVK**F**SRSPKILFVGDN 361

B._salanitronis_26A ISYVLV**W**RNAREKENHFYA-------PYPGQV------------SEQ**D**FVK**F**YNDPKTLFAGDM 364

B._sp._3_1_23_26A VSYVLV**W**RNAREKEAHYYA-------PYPGQI------------SAL**D**FVE**F**YKHPKTIFVSDL 363

B._ovatus_CL03T12C18_26A VSYVLV**W**RNAREKEAHYYA-------PYPGQI------------SAL**D**FVE**F**YKHPKTIFVSDL 355

B._sp._D2_26A VSYVLV**W**RNAREKEAHYYA-------PYPGQI------------SAL**D**FVE**F**YKHPKTIFVSDL 355

RsMan26C **W**SF**FM**P**WY**NNYCI--------PE**G**SNPYNSLEF**W**KKTMSSSL**VI**TLDNM**P**GW------------ 330

Prevotella_bryantii_B14_AAC97596.1 **W**LN**FM**P**WY**GE----------------SMPSDE**WW**TKVMNENV**VI**T**R**DEINQ-NATYMEESAQSA 587

uncultured_bacterium_ADA62505.1 **W**GH**FM**V**WY**QGGQGS----------TDTMCSDD**WW**KD**A**MSSAN**VI**T**R**DKVVIPDVTSTIEDATDA 371

uncultured_bacterium_ABB46200.1 **W**GH**FM**V**WY**QG**E**QGS----------TDTMCSDD**WW**KD**A**MSSAN**VI**T**R**DKVVIPDVTSTIENATDA 369

B._cellulosilyticus_26B **W**S**WFM**P**WYD**A**E**DA-----------ETP**HAD**Q**AWW**KD**A**ME**Q**NF**VI**S**R**E**DLP**DM**K**----------- 425

B._salanitronis_26B **W**L**WFM**P**WYD**SDNS-----------TTP**HAD**QT**WW**ED**A**MS**Q**DY**VI**K**R**GEFK-------------- 324

B._ovatus_CL03T12C18_26B **W**TY**FM**P**WYDY**SR**TK**ELDSEA**F**TATS**H**RY**ADK**D**WW**VD**A**MS**Q**DY**VI**TRDQ**LPSFK**----------- 399

B._sp._3_1_23_26B **W**TY**FM**P**WYDY**SR**TK**ELDSEA**F**TATS**H**RY**ADK**D**WW**VD**A**MS**Q**DY**VI**TRDQ**LPSFK**----------- 375

**BoMan26B WAWFMTWYDYEVTKDTTAPVFNSGQHEHADKAWWNNAFGQPGVICRSDLPSFK----------- 360**

B._xylanisolvens_NLAE-zl-P352_26B **WAWFMTWYDYEVTKDTTAPVFNSGQHEHADKAWWNNAF**S**QPGVICRSDLPSFK**----------- 360

B._sp._D22_26B **WAWFMTWYDYEVTKDTTAPVFNSGQHEHADKAWWNNAF**S**QPGVICRSDLPSFK**----------- 360

.: * . ..

B._cellulosilyticus_26A NSLYK------------------------------ 375

B._xylanisolvens_NLAE-zl-P352_26A FELYK------------------------------ 366

BoMan26A FELYK------------------------------ 366

B._ovatus_CL02T12C04_26A FELYK------------------------------ 366

B._salanitronis_26A KSN-------------------------------- 367

B._sp._3_1_23_26A K---------------------------------- 364

B._ovatus_CL03T12C18_26A K---------------------------------- 356

B._sp._D2_26A K---------------------------------- 356

RsMan26C ----------------------------------- 330

Prevotella_bryantii_B14_AAC97596.1 VDNFGLGFNLGNT---------------------- 600

uncultured_bacterium_ADA62505.1 VKNMGLGWNLGNALDANNQQYHDATQDNYWGQQD- 405

uncultured_bacterium_ABB46200.1 VKNMGLGWNLGNALDANAQQYHDATQDNYWGQQDI 404

B._cellulosilyticus_26B ----------------------------------- 425

B._salanitronis_26B ----------------------------------- 324

B._ovatus_CL03T12C18_26B ----------------------------------- 399

B._sp._3_1_23_26B ----------------------------------- 375

**BoMan26B ----------------------------------- 360**

B._xylanisolvens_NLAE-zl-P352_26B ----------------------------------- 360

B._sp._D22_26B ----------------------------------- 360

**Figure S5** Sequence alignment of all GH26 pairs encoded by type I and type II PULs, as well the other sequences from subbranch *Bo*Man26B (Figure 9). Residues conserved with *Bo*Man26B are marked in bold.

**Table S1** Generated mutants of *Bo*Man26B, showing both DNA and protein sequences. The mutated codon or residue is highlighted in bold.

| W112A | DNA | CCATGGCATGCTCTTCCTCGTCCGGGGAAGACTTTCCGGCTCCGGAGTCGGAACCGGTGGACAATTCATTGATAAAGAAAGAACTTTGCACCGAAGGTGCATCCGTTGAGGCTAAGAAAGTGTATACATATCTGAGAAACTGCTGGGGGAGAAAAACTTTGAGCAGTACAATGGCGAACGTGACATGGAACGTCAATGAAGCAATATGGGTAAACCGTCAGACCGGGAAGTATCCTGCCATTGCCTGCTTTGATTACATGAACCTTCCCGCTTCACCGGCCGAC**GCA**ATTGATTATAATAAGATATCGGTTGTTGAAGACTGGTGGAATGCCGGAGGATTGGTTGCTGCTTGCTGGCATTGGAATGTGCCGGTCACGGAAAATAGCAGTGAGTATAAATGTATGATTAGTGAAACGGATTTTGACATCACCAAGGCATTGCAGGAGGGAACCCGGGAAAATGAAATCATTAAAGCCGATTTGGAAGAATTGGCGGGTTACTTACTGCTTTTGAAGCAGAAGAATATTCCTGTAATTTGGAGACCGTTGCACGAAGCTGCAGGCAAATGGTTCTGGTGGGGGAAAGATGCAGCTTCATACAAACGGCTCTGGAAACTCGTATATGAGACTTTCAAGCAGAAAGGGCTGAACAATCTTATCTGGGTATGGACTAGTGAAACGAACGACAGGGACTGGTATCCTGGTGATGCATACGTGGATATTATCGGACGGGACGTGTACCACAAGACCAGCGCTGCCGGACTGGCAACGGATTTTGATGCACTAAAAAAAGCTTTCCCGGACAAGCTGATTGCACTTAGCGAATGTGGTGATGTGGCAACCATAGACAAGCAACTGGCAGCCGGAGCCCAATGGGCATGGTTCATGACTTGGTATGATTATGAAGTGACAAAGGACACTACTGCGCCTGTTTTTAATTCCGGACAGCACGAACATGCCGACAAGGCTTGGTGGAACAACGCTTTCGGCCAGCCTGGTGTGATTTGCCGTAGTGATTTGCCTTCGTTTAAACTCGAGCACCACCACCACCACCAC |
| --- | --- | --- |
|  | Protein | MACSSSSGEDFPAPESEPVDNSLIKKELCTEGASVEAKKVYTYLRNCWGRKTLSSTMANVTWNVNEAIWVNRQTGKYPAIACFDYMNLPASPAD**A**IDYNKISVVEDWWNAGGLVAACWHWNVPVTENSSEYKCMISETDFDITKALQEGTRENEIIKADLEELAGYLLLLKQKNIPVIWRPLHEAAGKWFWWGKDAASYKRLWKLVYETFKQKGLNNLIWVWTSETNDRDWYPGDAYVDIIGRDVYHKTSAAGLATDFDALKKAFPDKLIALSECGDVATIDKQLAAGAQWAWFMTWYDYEVTKDTTAPVFNSGQHEHADKAWWNNAFGQPGVICRSDLPSFKLEHHHHHH |
| W112F | DNA | CCATGGCATGCTCTTCCTCGTCCGGGGAAGACTTTCCGGCTCCGGAGTCGGAACCGGTGGACAATTCATTGATAAAGAAAGAACTTTGCACCGAAGGTGCATCCGTTGAGGCTAAGAAAGTGTATACATATCTGAGAAACTGCTGGGGGAGAAAAACTTTGAGCAGTACAATGGCGAACGTGACATGGAACGTCAATGAAGCAATATGGGTAAACCGTCAGACCGGGAAGTATCCTGCCATTGCCTGCTTTGATTACATGAACCTTCCCGCTTCACCGGCCGAC**TTT**ATTGATTATAATAAGATATCGGTTGTTGAAGACTGGTGGAATGCCGGAGGATTGGTTGCTGCTTGCTGGCATTGGAATGTGCCGGTCACGGAAAATAGCAGTGAGTATAAATGTATGATTAGTGAAACGGATTTTGACATCACCAAGGCATTGCAGGAGGGAACCCGGGAAAATGAAATCATTAAAGCCGATTTGGAAGAATTGGCGGGTTACTTACTGCTTTTGAAGCAGAAGAATATTCCTGTAATTTGGAGACCGTTGCACGAAGCTGCAGGCAAATGGTTCTGGTGGGGGAAAGATGCAGCTTCATACAAACGGCTCTGGAAACTCGTATATGAGACTTTCAAGCAGAAAGGGCTGAACAATCTTATCTGGGTATGGACTAGTGAAACGAACGACAGGGACTGGTATCCTGGTGATGCATACGTGGATATTATCGGACGGGACGTGTACCACAAGACCAGCGCTGCCGGACTGGCAACGGATTTTGATGCACTAAAAAAAGCTTTCCCGGACAAGCTGATTGCACTTAGCGAATGTGGTGATGTGGCAACCATAGACAAGCAACTGGCAGCCGGAGCCCAATGGGCATGGTTCATGACTTGGTATGATTATGAAGTGACAAAGGACACTACTGCGCCTGTTTTTAATTCCGGACAGCACGAACATGCCGACAAGGCTTGGTGGAACAACGCTTTCGGCCAGCCTGGTGTGATTTGCCGTAGTGATTTGCCTTCGTTTAAACTCGAGCACCACCACCACCACCAC |
|  | Protein | MACSSSSGEDFPAPESEPVDNSLIKKELCTEGASVEAKKVYTYLRNCWGRKTLSSTMANVTWNVNEAIWVNRQTGKYPAIACFDYMNLPASPAD**F**IDYNKISVVEDWWNAGGLVAACWHWNVPVTENSSEYKCMISETDFDITKALQEGTRENEIIKADLEELAGYLLLLKQKNIPVIWRPLHEAAGKWFWWGKDAASYKRLWKLVYETFKQKGLNNLIWVWTSETNDRDWYPGDAYVDIIGRDVYHKTSAAGLATDFDALKKAFPDKLIALSECGDVATIDKQLAAGAQWAWFMTWYDYEVTKDTTAPVFNSGQHEHADKAWWNNAFGQPGVICRSDLPSFKLEHHHHHH |
| K149S | DNA | CCATGGCATGCTCTTCCTCGTCCGGGGAAGACTTTCCGGCTCCGGAGTCGGAACCGGTGGACAATTCATTGATAAAGAAAGAACTTTGCACCGAAGGTGCATCCGTTGAGGCTAAGAAAGTGTATACATATCTGAGAAACTGCTGGGGGAGAAAAACTTTGAGCAGTACAATGGCGAACGTGACATGGAACGTCAATGAAGCAATATGGGTAAACCGTCAGACCGGGAAGTATCCTGCCATTGCCTGCTTTGATTACATGAACCTTCCCGCTTCACCGGCCGACTGGATTGATTATAATAAGATATCGGTTGTTGAAGACTGGTGGAATGCCGGAGGATTGGTTGCTGCTTGCTGGCATTGGAATGTGCCGGTCACGGAAAATAGCAGTGAGTAT**AGT**TGTATGATTAGTGAAACGGATTTTGACATCACCAAGGCATTGCAGGAGGGAACCCGGGAAAATGAAATCATTAAAGCCGATTTGGAAGAATTGGCGGGTTACTTACTGCTTTTGAAGCAGAAGAATATTCCTGTAATTTGGAGACCGTTGCACGAAGCTGCAGGCAAATGGTTCTGGTGGGGGAAAGATGCAGCTTCATACAAACGGCTCTGGAAACTCGTATATGAGACTTTCAAGCAGAAAGGGCTGAACAATCTTATCTGGGTATGGACTAGTGAAACGAACGACAGGGACTGGTATCCTGGTGATGCATACGTGGATATTATCGGACGGGACGTGTACCACAAGACCAGCGCTGCCGGACTGGCAACGGATTTTGATGCACTAAAAAAAGCTTTCCCGGACAAGCTGATTGCACTTAGCGAATGTGGTGATGTGGCAACCATAGACAAGCAACTGGCAGCCGGAGCCCAATGGGCATGGTTCATGACTTGGTATGATTATGAAGTGACAAAGGACACTACTGCGCCTGTTTTTAATTCCGGACAGCACGAACATGCCGACAAGGCTTGGTGGAACAACGCTTTCGGCCAGCCTGGTGTGATTTGCCGTAGTGATTTGCCTTCGTTTAAACTCGAGCACCACCACCACCACCAC |
|  | Protein | MACSSSSGEDFPAPESEPVDNSLIKKELCTEGASVEAKKVYTYLRNCWGRKTLSSTMANVTWNVNEAIWVNRQTGKYPAIACFDYMNLPASPADFIDYNKISVVEDWWNAGGLVAACWHWNVPVTENSSEY**S**CMISETDFDITKALQEGTRENEIIKADLEELAGYLLLLKQKNIPVIWRPLHEAAGKWFWWGKDAASYKRLWKLVYETFKQKGLNNLIWVWTSETNDRDWYPGDAYVDIIGRDVYHKTSAAGLATDFDALKKAFPDKLIALSECGDVATIDKQLAAGAQWAWFMTWYDYEVTKDTTAPVFNSGQHEHADKAWWNNAFGQPGVICRSDLPSFKLEHHHHHH |
| K149A | DNA | CCATGGCATGCTCTTCCTCGTCCGGGGAAGACTTTCCGGCTCCGGAGTCGGAACCGGTGGACAATTCATTGATAAAGAAAGAACTTTGCACCGAAGGTGCATCCGTTGAGGCTAAGAAAGTGTATACATATCTGAGAAACTGCTGGGGGAGAAAAACTTTGAGCAGTACAATGGCGAACGTGACATGGAACGTCAATGAAGCAATATGGGTAAACCGTCAGACCGGGAAGTATCCTGCCATTGCCTGCTTTGATTACATGAACCTTCCCGCTTCACCGGCCGACTGGATTGATTATAATAAGATATCGGTTGTTGAAGACTGGTGGAATGCCGGAGGATTGGTTGCTGCTTGCTGGCATTGGAATGTGCCGGTCACGGAAAATAGCAGTGAGTAT**GCA**TGTATGATTAGTGAAACGGATTTTGACATCACCAAGGCATTGCAGGAGGGAACCCGGGAAAATGAAATCATTAAAGCCGATTTGGAAGAATTGGCGGGTTACTTACTGCTTTTGAAGCAGAAGAATATTCCTGTAATTTGGAGACCGTTGCACGAAGCTGCAGGCAAATGGTTCTGGTGGGGGAAAGATGCAGCTTCATACAAACGGCTCTGGAAACTCGTATATGAGACTTTCAAGCAGAAAGGGCTGAACAATCTTATCTGGGTATGGACTAGTGAAACGAACGACAGGGACTGGTATCCTGGTGATGCATACGTGGATATTATCGGACGGGACGTGTACCACAAGACCAGCGCTGCCGGACTGGCAACGGATTTTGATGCACTAAAAAAAGCTTTCCCGGACAAGCTGATTGCACTTAGCGAATGTGGTGATGTGGCAACCATAGACAAGCAACTGGCAGCCGGAGCCCAATGGGCATGGTTCATGACTTGGTATGATTATGAAGTGACAAAGGACACTACTGCGCCTGTTTTTAATTCCGGACAGCACGAACATGCCGACAAGGCTTGGTGGAACAACGCTTTCGGCCAGCCTGGTGTGATTTGCCGTAGTGATTTGCCTTCGTTTAAACTCGAGCACCACCACCACCACCAC |
|  | Protein | MACSSSSGEDFPAPESEPVDNSLIKKELCTEGASVEAKKVYTYLRNCWGRKTLSSTMANVTWNVNEAIWVNRQTGKYPAIACFDYMNLPASPADFIDYNKISVVEDWWNAGGLVAACWHWNVPVTENSSEY**A**CMISETDFDITKALQEGTRENEIIKADLEELAGYLLLLKQKNIPVIWRPLHEAAGKWFWWGKDAASYKRLWKLVYETFKQKGLNNLIWVWTSETNDRDWYPGDAYVDIIGRDVYHKTSAAGLATDFDALKKAFPDKLIALSECGDVATIDKQLAAGAQWAWFMTWYDYEVTKDTTAPVFNSGQHEHADKAWWNNAFGQPGVICRSDLPSFKLEHHHHHH |

**
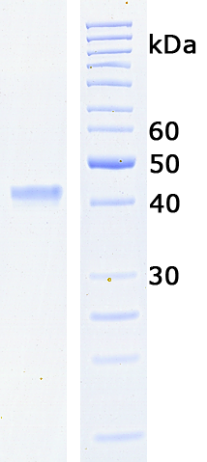
**

**Figure S6** SDS-PAGE of purified *Bo*Man26B, the ladder shown to the right with the sizes of relevant bands labeled in kDa. The gel has been spliced to remove inappropriate concentrations.
